# Guided Wave Propagation in a Realistic CFRP Fuselage Panel: Proof of Concept for Early-Stage Damage Detection

**DOI:** 10.3390/s25041104

**Published:** 2025-02-12

**Authors:** Fatma Sellami, Vittorio Memmolo, Mirko Hornung

**Affiliations:** 1 Department of Aerospace and Geodesy, Institute of Aircraft Design, School of Engineering and Design, Technical University of Munich, Boltzmannstr. 15, D-85748 Garching, Germany; mirko.hornung@tum.de; 2 Aerospace Structures and Materials Laboratory, Department of Industrial Engineering, University of Naples Federico II, Via Claudio 21, 80125 Naples, Italy; vittorio.memmolo@unina.it

**Keywords:** sensor measurements, ultrasonic waves, experimental set-up design, aeronautical composite structure, digital signal processing, sensor fusion, health indicators

## Abstract

This paper presents an experimental study of wave motions and a global diagnostics method in a realistic aerospace-grade composite component with a complex design. Due to the frequency dependence of the velocity, wave propagation in anisotropic materials is difficult to describe quantitatively. The analysis of experimental ultrasonic wave propagation and the interactions with discontinuities in thin-walled aircraft structures can provide a plethora of information on the wave structure, the mode shapes, and stiffness reduction. An experiment is devised with a network of various omnidirectional sensor configurations to activate and measure structural responses. The measurement process can be leveraged for flaw detection in large multilayered structures. Physically, this corresponds to analyzing the dispersive behavior and scattering properties of ultrasonic waves, the shape of the waveforms, and their corresponding velocities. Ultrasonic waves are measured in a realistic CFRP fuselage panel in a pristine state and after impacts at different energy levels. Simulations do not allow the wave motion in complex and large design structures to be entirely comprehended. The sensitivity of the guided waves as a damage detection tool is proved for the fuselage structure by an extensive measurement campaign and a probabilistic imaging approach based on health indicator fusion.

## 1. Introduction

Lightweight design in aviation is related to weight and fuel consumption reduction, CO_2_ emissions, and improved aerodynamic efficiency throughout the various flight phases. To achieve better performance, composite materials, especially carbon fiber-reinforced polymers (CFRPs), have increasingly come to the attention of aircraft manufacturers. The first use of CFRP in the primary structures of a commercial aircraft started with the A300. The main advantage was a weight reduction of 20% in comparison to aluminum and cost savings in the assembly work. Boeing B787 Dreamliner and Airbus A350 XWB are, respectively, 50 and 53 percent composite by weight. In the former, 1500 aluminum sheets and 40,000–50,000 fasteners per section were eliminated through composite integration [1,2,3,4].

In the A380, the following parts are made of lightweight composite materials: the horizontal and vertical tail pan, J-nose, floor beams for the upper deck, outer flaps, vertical tail plane, the rear pressure bulkhead, wing ribs, and center wing box. In the helicopter field, the use of composite material also increased between 1967 and 2015. In the beginning, only the rotor blades and various cladding components were made of composite material, but then CFRP was increasingly used for the airframe and the tail boom. From 1990, modern helicopters in the civil sector were up to 80% CFRP, and military helicopters 80% to 90% CFRP (e.g., the Tiger and NH90) [5].

The typical properties of commercial carbon fibers and the airframe materials distribution in Boeing 787 are reported in [1,6]. Ref. [6] also reports the typical CFRP aerospace components formed by several techniques (i.e., laminating for shells for fuselage section sheets, integrally stiffened for wing skins and empennage skins; beams for spars and ribs; filament winding for closed shells like pressure vessels; open shells for rocket motors, secondary formed tubes for the helicopter blade; braiding for fuselage frames, propellers and helicopter blades; pultrusion for beams such as stringers, floor beams, spars, ribs, and longerons). The advantages and disadvantages of CFRPs compared to aluminum alloys are also reported in [6], along with the non-destructive inspection (NDI) methods for detecting barely visible impact damage (BVID).

The inclusion of composites in the material selection in both commercial and military airplanes means lower operating costs over the life of the airplane. The optimal material composition according to the structural design enabled a new design paradigm based on tailored structures that provide higher mechanical performance, lower thermal expansion, high material damping, corrosion resistance, and electromagnetic insulation. Stacking layers with fibers at different angles is often performed to lessen the anisotropy of the laminate, which is a few millimeters thick. Nevertheless, the anisotropic properties of the materials and the lack of surface visibility for a variety for damage occurring during routine maintenance operations or flights make the maintenance process a more challenging task to perform than with metal components.

Damage occurring through the entire volume and showing no change in the physical and geometrical properties can propagate and stay undetected until a dangerous outcome worsened by environmental conditions occurs. For this reason, a damage tolerance approach based on accommodated material allowables (limit stress–strain reduced to compensate for the presence of hidden damage) and continuous inspections is in use among aircraft manufacturers and airlines.

Among various non-destructive inspections, those based on ultrasound are quite efficient and reliable [7,8], paving the way toward more general monitoring strategies using permanent transducers [9]. When using ultrasonic wave analysis as a structural monitoring approach, a thoughtful understanding of wave motion in real-scale aeronautical components is necessary to assess the complexity of the experimental data and the necessary tools for their generation and processing. The complexity of the material and structural response makes mechanical structural modeling and experimental investigations more expensive and complicated in comparison with isotropic materials [10]. In the current literature, experimental data from ultrasonic testing are obtained from simple uniaxial, pure shear tests and impacts on large metallic and small composite structures. In addition, very limited data are available for large aeronautical composite structures [11,12,13,14].

In this context, this study contributes by providing experimental data and insight into the characteristics of Lamb waves propagating in a complex laminated fiber-reinforced and stiffened composite structure. Testing complex non-generic designs and providing insight into more test data allow us to better prepare for the transition from simple plates, extensively analyzed so far, to complex structures that have not been thoroughly investigated yet, exploiting the principle of the building block approach adopted in aeronautics [15].

As a general trend, piezoelectric active sensors have been employed on a massive scale to deal with ultrasound thanks to their light weight and long durability. Indeed, they can be employed to generate and evaluate the propagation and scattering of elastic waves in thin-walled structures such as those found in the aerospace industry. Aircraft components such as wing skins, empennages, fuselage skins, gas turbine blades, and combustor castings in aero-engines are possible candidates to be inspected with the help of guided waves. A recurrent problem encountered during the maintenance of aircraft structures is the inaccessibility of some components. Dismantling parts to assess their health state becomes a risky task that might itself result in structural damage. Therefore, the implementation of guided waves can be a possible solution if sufficient reliability is achieved in detecting damage.

To this end, the present study aims to develop a method that can continuously predict the degree of degradation in load-bearing and stiffening members by using permanently installed ultrasonic transducers. The use case of this study is a component from a rear-side shell fuselage structure. The wave motion in the material and the generation of guided waves as well as their interaction with discontinuities and structural designs are analyzed for different sensor configurations. The guided wave signal is dispersive and contains a multitude of modes interfering and traveling at different phase and group velocities with significant coherent noise that cannot be consistently averaged out. One way to deal with the dispersive behavior issue is to activate the fundamental non-dispersive modes, namely the symmetric in-plane mode S0, the antisymmetric out-of-plane mode A0, and the shear horizontal mode S0’.

Despite the extensive literature on the use of dispersive ultrasonic guided waves for damage detection, there is still a lack of demonstration of this structural health monitoring (SHM) approach on real-scale structures, especially in addressing key challenges.

- Signal processing is complicated by complex geometry. While stiffeners, thickness variations, cutouts, and other geometrical discontinuities are integral to the design of aerospace structures, most SHM approaches are demonstrated on simplified, laboratory-scale structures. In contrast, the investigated structure is characterized by the skin area of the thin shell, with slightly varying thicknesses across the entire surface. The thickness of the laminate, the size of the panel, the presence of the six omega-shaped stringers, and the boundary conditions are all factors that influence both the impact dynamics and wave propagation [16];
- When many transducers are adopted, electromagnetic noise is exacerbated, making it more difficult to properly detect signal variations caused by damage;
- There is insufficient evidence of damage detection in real-scale structures where the symmetric mode is used and activated by a one-sided excitation setup, the only feasible configuration for real-world applications. This approach is particularly advantageous at relatively high frequencies because the wavelength becomes shorter, increasing the sensitivity of ultrasonic waves to very small flaws. This is crucial for realistic fuselage segments, which are load-bearing structures subjected to relatively low air loads but large concentrated forces from wing reactions, tails, landing gear, and payloads [17]. Consequently, detecting very small emerging flaws is vital, as they could rapidly grow under large forces coupled with fatigue loads.

In the context of the previous item, a significant difference exists in the basic morphology of impact-induced damage between simulated damage (e.g., using an insert between laminae or reversible damage that alters wave propagation) and impact-induced damage replicating actual conditions occurring during the structure’s lifetime. Indeed, damage initiated by transverse impact loads on thin-walled composite laminates can lead to fiber fracture and matrix cracking. These matrix cracks, often arranged in a complex pattern, induce delamination at the interfaces between plies with different fiber orientations. Such flaws can cause a dramatic reduction in laminate compressive strength, leading to unexpected failure of the thin-walled composite structure due to microbuckling of fibers or local buckling of plies [16,18]. Nonetheless, most studies in the literature use either reversible damage (e.g., metallic mass bonded to the structure surface) or artificial damage (e.g., Teflon patch discontinuity). While both damage types affect wave propagation, the result is significantly different. Additionally, barely visible damage is often present when impact-induced damage occurs. This study aims to keep the impact energy as low as possible to demonstrate early damage detection capabilities, particularly when the damage is invisible.

This paper addresses these issues related to the implementation of SHM in a real-scale fuselage segment, demonstrating the viability of guided wave (GW)-based SHM for aerospace primary structures. The scope of this study is threefold: (1) devising an experimental procedure for large stiffened aircraft components and performing a test campaign; (2) evaluating the wave velocity variations and the propagation of stress waves excited with various piezoelectric sensors in real structures in pristine and damaged states; and (3) calculating damage indices and the fusion of those to detect and locate barely visible damage in a real large aircraft component.

The following research points are investigated:

- Developing a measurement design set-up scheme for collecting wave signals in a composite primary structure, a fuselage frame.
- Measuring and analyzing the velocities of Lamb waves propagating in an aircraft structure.
- Analyzing a large amount of experimental ultrasonic data collected from a realistic structure throughout a two-year test campaign to identify random and systematic errors in both the instrumentation and analysis processes. And optimizing the set-up design accordingly.
- Applying a tomographic imaging reconstruction algorithm and validating it for a complex aircraft component instrumented with sparsely distributed piezoelectric sensors.
- Providing insights into the assessment of the structural integrity of complex structures for which information on the physical properties and maintenance records is limited or unknown.

## 2. Background

Elastic wave motion in fiber-reinforced composite structures shows strong angular dependency. Depending on the structure’s lay-up, the phase and group velocities of guided waves are different and dispersive, i.e.,varying with the frequency at which they are being actuated and measured [19].

On a broader concept, technological approaches that have been investigated with the purpose of establishing a global monitoring system are the optical fibers-based systems (e.g., the fiber bragg grating [FBG], the optical frequency domain reflectometry [OFDR] [20,21,22,23]) and systems relying on acoustic and ultrasonic waves such as acoustic emission and Rayleigh–Lamb waves [24,25,26]. Surface acoustic wave (SAW) sensor technology is, for instance, a local method which consists of an electrical signal in the form of chirp changed into mechanical waves by an interdigital transducer (IDT). The SAW sensors, investigated at the NASA Langley Research Center, can measure various quantities such as strain, temperature, and loads [27,28]. The SAW strain sensors are also used for the detection of aircraft fastener failures [29,30].

The theory of Lamb waves was first introduced in 1916 by Lord Lamb [31] and later experimentally proved in the work of Viktorov [32] in 1967. Their use to detect flaws in plate-like structures has been widely investigated in the literature [33]. However, their application for realistic aerospace components with complex designs is rather limited. The challenge relies on the wave structure and specifically when interacting with discontinuities during propagation in large multilayered structures, as it generates difficult-to-interpret time-domain signals with a multitude of wave packets. Nonetheless, continuous monitoring of elastic wave propagation allows both local and global damage monitoring. In particular, the use of networks of piezoelectric sensors generating several wave paths has been found to be well suited for inspecting thin plates like those found in aircraft structures [34].

The very first developed commercial structural health monitoring (SHM) system based on Lamb waves was produced at Standford University and consisted of so-called SMART Layers. The 6.5 mm in diameter piezoelectric sensors integrated into conductive material layers can be applied to stiffened composite structures [35]. The purpose of such sensor layers is also to integrate them into the composite structure during manufacturing, but such integration has not yet been fully mastered or proven applicable and reliable. In Ref. [36], an electromagnetic technology named HELP (hybrid electromagnetic performing layer), based on the local measurement of electrical and magnetic fields, was developed at the French Aerospace Lab (ONERA). It is mainly efficient at detecting damage-inducing variations in electric properties. On the other hand, acousto-ultrasonic methods are more sensitive to mechanical defects (for instance, damage that does not cause fiber breakage, the conductive part of the composites). The combination of both SMART and HELP layer technologies provided an improvement in damage detection in simple carbon/epoxy plates [37].

Wave velocity estimation is quite important in characterizing Lamb wave propagation in plate-like structures as it defines its dispersive behavior as a function of frequency. However, one of the main challenges for measuring the group velocity of Lamb waves is to extract the exact onset time of the wave packets accurately. Usually, for accurate estimation, two receivers and a time correction are needed. In Ref. [38], a calibration procedure to extract the group wave velocity was proposed. In the latter, the signal–energy-based algorithm is used for damage detection. A time correction related to the maximum value of the excitation pulse was applied in [39].

Elastic waves are sensitive to defects to different degrees. According to some studies, antisymmetric modes proved to have higher sensitivities to non-linear defects like cracks and disbonds than to linear defects like voids. The selection of modes could be made by a strategic sensor placement, the choice of actuation frequency, and the design of the sensors [40,41,42,43].

To perform damage detection and localization, damage indices need to be calculated [44,45].

Tomographic systems typically rely on either the time-of-flight or the amplitude information to generate an image located within a polygon bounding the ultrasonic transducers [46]. Timeshift averaging algorithms were applied to differential signals filtered at multiple frequencies, resulting in many images for the same structural state, and these images were fused to improve damage localization and background noise [47]. In Ref. [38], the delay-and-sum algorithm was applied for damage localization. The damage is in the form of holes and notches, and the complexity of the 4000μs signal constitutes an interesting example of experimental data. Even for simple plates, the signal has multiple wave packets that represent the mode shapes of the structure, its design details, and the discontinuities present within the material.

The sum and delay algorithm where the envelope-detected differential signal is the input when working on composites needs to take into consideration the fact that the propagation angles are different, and thus the group velocity needs to be adjusted according to the radial pattern of velocity.

However, many of those algorithms introduce artifacts that may alter the damage reconstruction.

The minimum variance distortionless response (MVDR) beamforming [48], also known as Capon’s method, can be applied to minimize the artifacts that appear as reflections and disrupt the damage detection process. This offers an improvement over the delay-and-sum algorithm.

Further imaging methods are possible, but their use has mainly been proven on small structures [46,49]. Larger structures, such as a 1 m × 1 m CFRP composite plate with artificial holes and crack-type defects, were investigated in [35]. The total focusing method (TFM) with full matrix capture was applied to guided wave signals. The panel does not include stringers and is flat, which makes it more suitable for Lamb wave propagations. In Ref. [50], shear mode transducers were used to assess bondline integrity in a laminate structure under three-point bending.

The use of guided wave-based SHM systems on real-scale structures is rather limited and needs further attention on the way to implement ultrasonic waves in a reliable manner, accounting for the structural complexities other than the anisotropic behavior.

## 3. Safety Standards in Aircraft Design and Airworthiness Management

The European Union Aviation Safety Agency (EASA) document Easy Access Rules for Large Aeroplanes Certification Specification (CS)-25 [51] states the requirements for performing non-destructive testing (NDT) and for assessing the defect types that need to be monitored and addressed. This ensures the techniques best suited for specific maintenance tasks can be identified and calibrated. Defects that do not endanger the flight must be monitored and recorded in the aircraft-maintenance record system and rectified as soon as possible. NDE techniques for detecting defects in composite materials are reported in [52] from NASA Technical Memorandum 4322A, NASA Reliability Preferred Practices for Design and Test [53]. It states that delaminations, voids, micro-cracks, and wrinkles can be detected using the traditional ultrasonic method. The investigated use case is classified as a primary structure that carries flight, ground, or pressurization loads and whose failure would reduce the aircraft’s structural integrity [54]. To detect damage of length *L*, a wavelength equal to or below 2×L needs to be used [55]. In acoustic and ultrasonic measurements, if the chosen wavelength is too large in comparison with the flaw size, the wave further propagates in the structure with limited interactions with the defects. However, by using opportune signal processing, even minor damage can be detected. The following mathematical relation between the diameter of the sensors (${⌀}_{PZT}$) and the wavelength (λ) is not applicable in every case:

(1) $${⌀}_{PZT}\leq \frac{\lambda }{2}\leq L$$

with *L* being the damage length.

This paper deals with detecting Category 1 damage (barely visible impact damage [BVID]) and allowable defects caused in manufacturing or service, as defined in [56].

### 3.1. Composite Damage-Tolerance Approach

Composite materials are widely employed in aeronautics to design lighter and stiffer load-bearing structures. Indeed, they allow structural properties to be tailored according to the actual load with an optimal compromise between weight and strength/stiffness. Furthermore, manufacturing in a one-piece barrel offers a unique weight-saving possibility, as connections are not needed.

Nonetheless, composite materials have a number of drawbacks preventing their full exploitation, thereby offsetting theoretical benefits. In this regard, one of the greatest concerns is barely visible or invisible damage due to random events, such as certain low-velocity impacts. To accommodate the negative effect of impact-induced damage, stiffened composite laminates are subjected to overdesign and strict maintenance schedules. This results in operational costs at the aircraft level increasing greatly, which reduces or even cancels out the positive impact of using composite materials. Consider, for instance, the fracture mechanics of composites. After the surface has been impacted by external objects involving a few joules of energy, the interfaces between adjacent plies with different material orientations may separate. Although this reduces stiffness significantly, the hidden flaw is complemented by a small external indentation and cannot be detected even when resorting to detailed visual inspection [57].

In recent decades, the scientific community has focused on enhancing knowledge about the mechanics of failure for composite aircraft structures. Nonetheless, the detectability of damage remains a major concern. Impact-induced bending or shear stresses can generate micro-cracks in the matrix at the laminate side opposite to the impact side. These micro-cracks propagate back to the nearby interface, yielding delamination between dissimilar plies due to very high interlaminar shear stresses. This can lead to significant and distributed through-thickness failure, which is barely visible from the outer surface [45]. However, though damage conformation can be easily assessed by non-destructive inspection, the external surface only shows indentations of a few hundred microns, making early detection challenging. For composites, to prevent this unpredictable behavior from triggering damage onset and unstable flaw propagation, a specific design is followed by aeronautical manufacturers.

The damage-tolerance practice employed in designing modern aircraft relies on several factors: residual-strength information, the prediction of damage-growth behavior and environmental degradation, the scheduling of maintenance tasks, and prior confidence in damage experienced during lifetime.

To ensure these are met, a type of “defect factor” based on the degree of detectability is introduced to establish minimum damage-tolerance residual strengths for composite structures [58]. Damage-tolerance philosophy introduces knockdown factors that reduce composite material strength to neglect complex impact mechanics. However, this results in unpredictable structure behavior when bearing loads. Indeed, hidden flaws drastically reduce fatigue and compression strength (usually referred to as compression after impact [CAI] strength), both of which are critical design parameters. Generally, CAI strength may be reduced to around 60% of the pristine value. Likewise, the loss of fatigue strength can reach 70% of the pristine value, depending on the fatigue-load spectrum and the laminate lay-up [59]. In this sense, the damage-tolerance approach is accomplished by limiting the material’s design strain level in the design criteria [56]. As such, a compressive strain is usually limited to a value between 3000 and 4000 microstrains during design [60].

**Figure 1:**
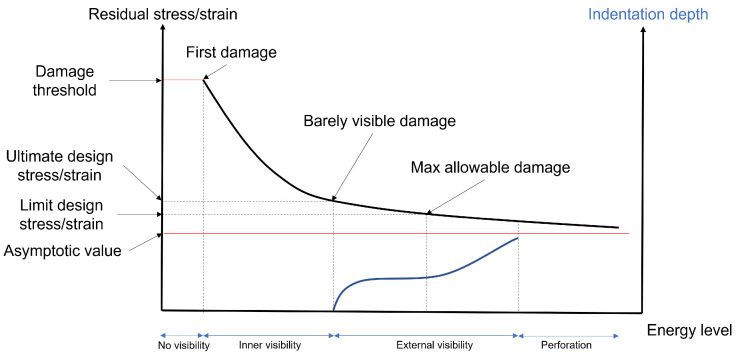
Schematic of residual strain versus energy level and the derived design constraints [63].

Benefits introduced by composite structures are further limited to preserve the bearing capability of reinforcements. In aerostructure design, stringers are adopted to enhance the stiffness of thin-walled structures at the lightest weight possible. However, in the case of delamination, low-energy impacts may cause the stringer separation from the host structure (disbonding), preventing structural collaboration and proper loading absorption [61]. To avoid unstable disbonding propagation, crack stoppers are generally introduced to limit the maximum damage size and allow safe structural collaboration [56]. As a matter of fact, connectors are necessary despite the opportunity of exploiting one-piece barrel manufacturing to reduce weight, and they have their own production and maintenance costs. This rough damage-tolerance approach, therefore, offsets the benefits of using composites. In principle, residual-strength capability and damage-growth characterization are the two primary damage-tolerance requirements described in [62]. In terms of the former requirement, the certification tests of composite structures pass through a compliance demonstration, which includes bearing design ultimate loads when damage at the threshold of visual detectability is present and withstanding design limit loads when visible damage (above the threshold) is present. In terms of the latter requirement, it is necessary to demonstrate that small damage (i.e., not affecting the residual strength) does not experience detrimental growth under operational loads. To this end, visually undetectable damage must not grow under operational loads between two major adjacent inspection checks. For example, visible damage induced by foreign-object impact does not grow between two “C” checks (4000 flight hours per “C” check). To further illustrate how a damage-tolerance design is influenced by the occurrence of unforeseen events, Figure 1 shows a schematic of residual stress–strain trend versus the impact energy level. When the energy level is very low, no damage is present. This is because there is a threshold energy causing damage onset. When the first damage arises, the residual stress–strain shows a deep decrease, but the damage remains hidden. This is an example of through-thickness failure, either without any visible indentation (inner visibility) or with a slightly visible indentation (external visibility). In any case, this damage cannot be reliably detected by visual inspection. A further increase in energy leads to increasingly observable damage and a reduced stress–strain rate decrease. This constitutes the limit where the ultimate design stress–strain is set.

**Figure 2:**
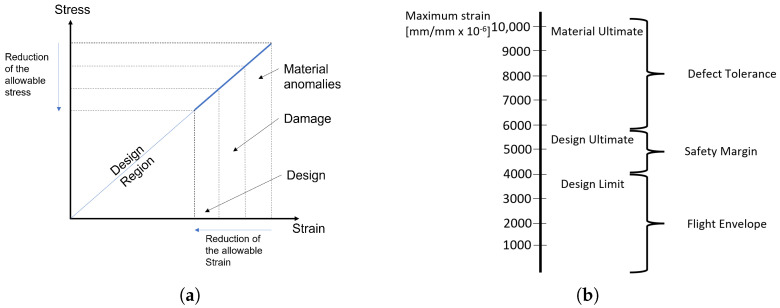
Schematic of scattering factors constraining the composite design (**a**) and typical design stress–strain allowables for CFRP (**b**) [63].

Nonetheless, the damage is not the only cause of knockdown factors. Other aspects demanding attention during design are shown in Figure 2a. This scheme depicts the allowable design region after reducing the allowable stress–strain due to knockdown factors. Due to the introduced tolerance, composite materials are sized using limit allowables appreciably lower than the material ultimate allowable (see Figure 2b). Both Boeing design manuals and military handbooks [64] offer a rough estimation of the design limit allowable $\sigma_{d.l.a.}$, where they halve the ultimate material allowable $\sigma_{m.u.a}$:

(2) $$\sigma_{d.l.a.}=0.5\sigma_{m.u.a}$$

A damage-tolerance investigation was performed at the Netherlands Aerospace Centre (NLR) and reported in [65]. The effects of impacts on CFRP structures and examples of the detection difficulties encountered with BVID are reported by [6].

### 3.2. SHM Perspective in Aircraft Design

The previous section shows how the weight saving expected from introducing composite materials is wasted in implementing a damage-tolerance approach. However, introducing damage-monitoring capabilities can lead to a less strict design. Indeed, the allowable stress–strain can be set to the residual stress–strain associated with the minimum detectable damage that the SHM system can reliably identify. Future airliners could have even lighter composite structures if new lessons on structural health data are applied. Indeed, the material design could be less constrained. Current aircraft such as Boeing 787 or Airbus A350 are “structurally overdesigned,” and their successors could lose weight without compromising structural integrity. Nonetheless, the standardization and reliability required to move from a damage tolerance to a condition-based design make it challenging to integrate SHM technology during structural design. For these reasons, SHM-based design can be referred to as long-term perspective of SHM. The short-term perspective of SHM is associated with the damage-assessment procedure. A condition-based maintenance philosophy can reduce the number of maintenance tasks (being limited to monitoring system warnings), minimizing aircraft downtime, and reducing the direct operating costs of an aircraft significantly [66]. This latter perspective is made possible by the inherent capability of an SHM system permanently integrated into the structure to perform on-demand inspections.

Unlike NDE, these inspections are always carried out in the same way, thereby allowing the development of a historical database that includes updated information about structural health and can assist in such a reasoning system. However, to this end, advanced data analytics is required to identify characteristic material changes and update new residual life according to the information gathered from the monitoring system and scaled up to a prognosis level. The idea behind this approach is that key features extracted from SHM raw data significantly change with the health condition and can be stored in a structural health database, which is updated at every SHM inspection. This monitoring process can be generally enhanced by combining existing non-destructive inspection (NDI)/non-destructive evaluation (NDE), whose results populate the historical database.

In addition, prognosis techniques make use of linear assumptions, which often do not match the mechanics of composites. Hence, the densified SHM data populated by NDI outcomes can be used to compensate for the prediction errors in residual life estimation and adjust the crack-growth prediction laws [67]. In this view, SHM can contribute to structural diagnosis and prognosis as well as maintenance scheduling by integrating the structure with the capability to monitor critical components.

All this flows in the conceptualization of an integrated health management system, shifting preventive (or scheduled) maintenance into more relaxed predictive (or on-condition) maintenance. Despite this promising perspective, condition-based philosophy demands an effective and reliable damage detection system, a damage onset warning, and a reasoning system. This process is directed at analyzing the effects of the defect initiation and triggering maintenance workflows.

Integrating a network of transducers, collecting associated data, and processing them via data-analytics algorithms is a reverse engineering approach yielding damage diagnosis and prognosis [61]. For this reason, it is worth cultivating knowledge of the adopted approach through an extensive experimental campaign of real-scale aircraft structures, which is the main motivation behind this work.

## 4. Experimental Design

A principal use case is treated in this study: A multilayered fiber-reinforced composite structure with six omega-shaped stringers. More precisely, a realistic CFRP fuselage panel with the following dimensions is considered: 1260 mm × 1150 mm × 35 mm. The last dimension includes the height of the stiffeners used to reinforce the shell structure.

The thickness of the structure was measured along the 1.26 m length using an outside micrometer on 10 spots that were polished to remove paint and corrosion treatments. It was found to be equal to 2.36 mm (SD = 0.02, CV = 0.84%). There were some variations in the thickness of the whole structure.

**Figure 3:**
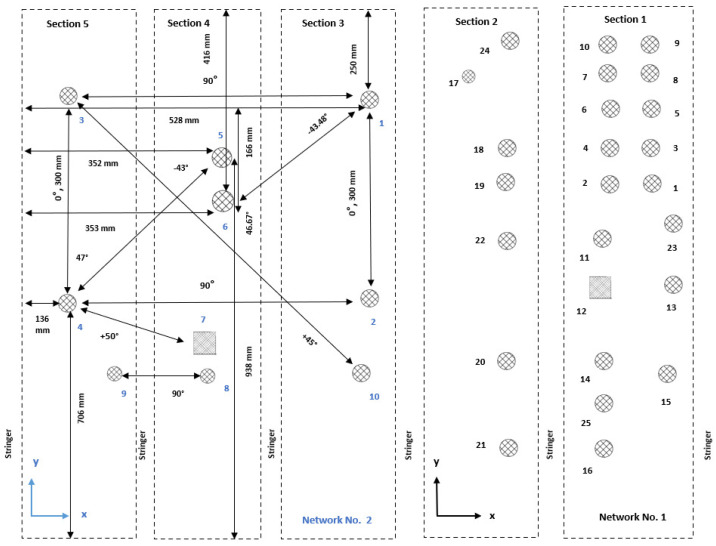
Sensor networks adopted for SHM. The numbers (in black for Network No. 1 and in blue for Network No. 2) identify the sensors.

An experiment was devised to excite and measure elastic waves propagating in a large multilayered structure using both pitch-catch and pulse-echo modes. In the former modality, one transducer is excited, and the remaining sensors record waves propagating across their location. The latter mode relies on the use of the same transducer as the actuator and receiver of elastic waves. In this way, any echo scattered back to the actuator is recorded. To cover the whole component, different networks of sparse transducers are integrated on the surface of the structure. The sensor configurations of such networks are shown in Figure 3. Details about the sensor types are available in Appendix B.

Even though the piezoelectric elements’ placement could be equidistant, the setup was intentionally arranged differently. The reason is connected to replicating a real case scenario, as many constraints could prevent a homogeneous sensor density. For this reason, in network No. 1, the transducers were optimally placed according to the stiffeners’ location to maximize coverage. Instead, in network No. 2, the transducers were minimized to check the minimum number of transducers needed for damage detection and localization.

Different transducer clusters (using sensors from Physik Instrumente and Reichelt Elektronik) were designed to monitor the specified area. The first network consists of Sections 1 and 2. The second network consists of Sections 3 to 5. There were additional sensors placed on the structure, but they were used for the wave propagation analysis rather than for the damage detection process, which is limited in this study to the two networks displayed in Figure 3. In Sections 3 and 5, a positioning laser was used to measure the distances between the sensors and the distances from the sensors to the structure’s edges. The measurement allowed the calculations of the angles between Sensors 1 and 6 and between Sensors 5 and 4.

The aim was to place sensors in −45° and +45° directions as accurately as possible. However, because the distance between the stringers was only 130 mm (which is, in certain cases, too short a distance to obtain a good waveform where the first arrival wave packet is well separated from the electromagnetic noise), it was only possible to realize the $\pm 45^{\circ }$ configuration on this face of the panel when positioning the second sensor for every sender–receiver pair across the stringers. Therefore, Sensors 5 and 6 were placed in Section 4. The gluing process caused uncertainties in the positioning of the sensors. The angles were not exactly equal to 45°, but this was sufficient to assess the wave propagation in this direction with an error of 2°. After careful placement and gluing with epoxy, the obtained angles between Sensors 1 and 6 and 5 and 4 were equal to −43.5° and −43°, respectively.

The angles between Sensors 1 and 2 and Sensors 3 and 4 were equal to 0°, and the angles between Sensors 1 and 3 and Sensors 2 and 4 were equal to 90°. In Sections 3 to 5, the wave propagation as well as the damage detection processes were investigated. Sensors 1 to 6 in these three sections had the same dimension (10 mm × 0.5 mm). It is worth pointing out that the shape of the transducer does not affect wave propagation [68].

**Figure 4:**
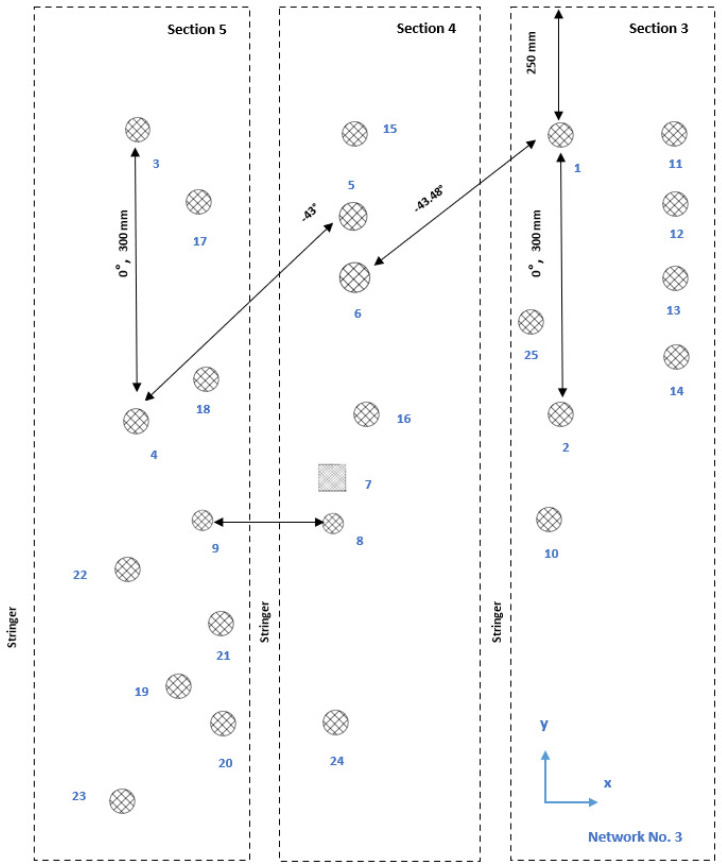
Network No. 3: Network No. 2 with a higher sensor coverage. The numbers identify the sensors.

The first damage detection computations proved that a network composed of 10 sensors was not enough for the localization of barely visible damage. Then, this experimental design was adjusted to achieve a higher sensor density. On that count, a third network was designed with a higher sensor coverage (i.e., 25 sensors, as for Network 1). More sensors were specifically placed in the area where the multiple impact damage was generated. This allowed more paths to cross the damaged area and allowed the sensor density to be correlated to the Lamb wave sensitivity in detecting barely visible damage in a stiffened CFRP structure. This sensor configuration is shown in Figure 4.

The panel was used to collect data, assess the gluing capabilities and sensor states over time, and assess wave propagations and interactions with discontinuities in a complex solid medium. The wave velocities in different propagation directions were experimentally measured using an active network of omnidirectional sensors. Stress waves were generated in the structure using a data acquisition system that integrates an arbitrary waveform generator and a power amplifier. The waveform generator allowed us to define an actuation amplitude signal of a maximum of 90 Vp-p. The actuation signal used to excite the piezoelectric sensors was a narrowband count burst excitation of 3 to 10 sine cycles with a center frequency varying between 70 kHz and 600 kHz. The digitizer had a fixed input range of ±1V. The analog sensor signals were bandpass filtered with a finite impulse response filter, digitized with a 12-bit resolution A/D converter, and sampled at an adjustable rate of 6 to 48 Mega samples per second. The system resolution was equal to 488 μV/bit (the A/D quantization error band was in the ±244μV range). The system had 32 channels that allow the definition of several propagation actuator–sensor routes. Every sensor could be set as both sender and receiver. Pitch catch and pulse echos signals were successively recorded between 100 kHz and 400 kHz. The signals were averaged 10 times to achieve a better signal-to-noise ratio. Filtering and amplification were parts of the signal conditioning process. Depending on the sensor response signal obtained at a particular actuation frequency, the circuitry gain (between 19 dB and 59 dB) was adjusted accordingly, allowing assessment of the wave velocity in signals with otherwise undetectable waveforms. For most of the experiments, a five-count burst signal was the selected actuation pulse, and the sampling frequency was equal to 48 MS/s.

**Figure 5:**
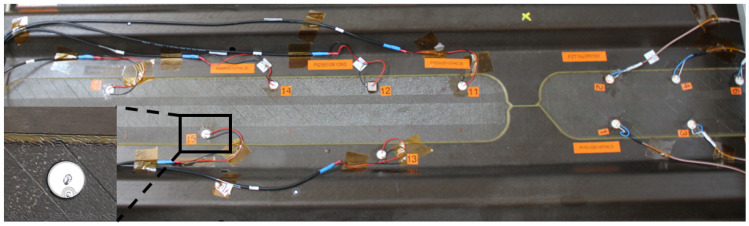
Example of a sensor network.

**Figure 6:**
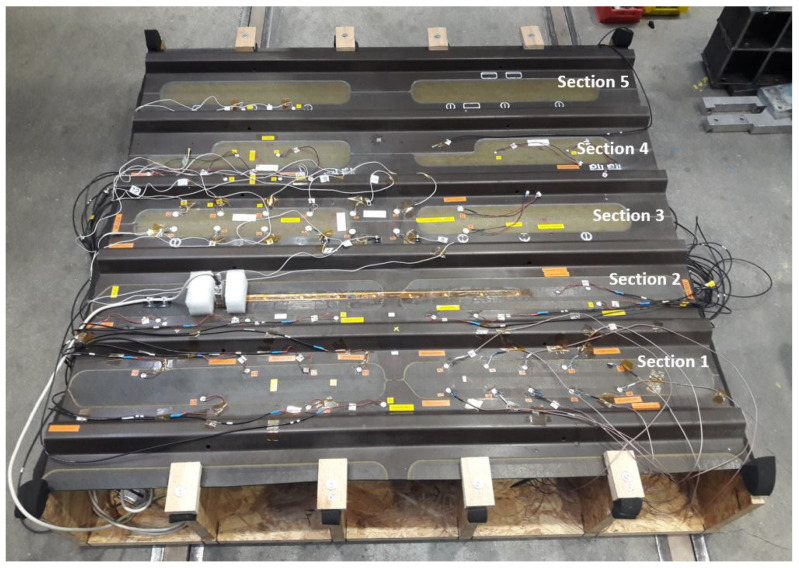
The investigated CFRP fuselage panel with the five sections.

The CFRP panel with the defined sections, before full sensor instrumentation, is shown in Figure 5 and Figure 6.

The burst actuation was carried out at 0% to 100% maximum voltage. When 20 V and 30 V were selected, 44% and 67%, respectively, of the maximum actuation voltage were applied. All measurements were performed at room temperature and directly after an impact was performed. Subsequent measurements were performed as well, days and even months later.

The measurements were performed in a big workshop hall. When performing damage detection, the baseline could be either the pristine state or a previous state of the structure.

Several propagation paths were implemented in-between and across the stiffeners, at different propagation angles, and on both sides of the fuselage structural section. On the reverse side of the composite panel, there was greater freedom of sensor placements; however, for real applications, this is not a viable option. The investigation shows the adjustment of the experiment parameters, explained above, to cover all the situations needed for the wave data collection.

**Figure 7:**
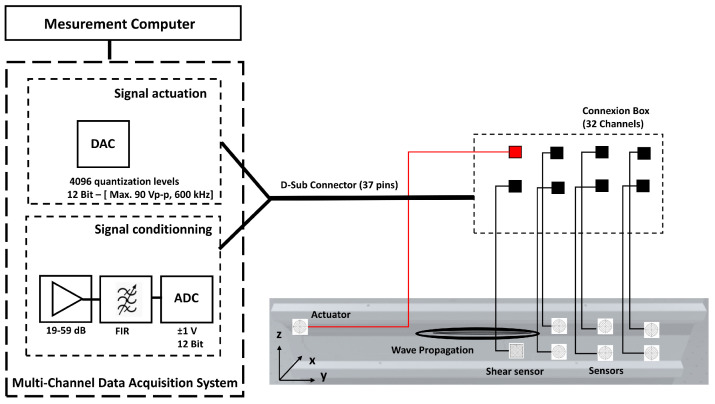
Data acquisition system.

The velocities of the first arrival wave packets were determined in all major directions defined by the propagation routes. The set-up to perform the guided wave measurements is shown in Figure 7.

Some ultrasonic measurements were performed on the same day of the impact tests and some others at a subsequent time, in successive sessions. The tests were performed at room temperature over 24 months. The temperature varied between 19°Cand23°C±2°C.

### 4.1. Impact Testing

The impacts were brought by a drop-weight impact tester (drop tower equipped with accelerometers from Kistler Instrument Corporation (Winterthur, Switzerland), PCB Piezotronics GmbH (Hückelhoven, Germany), and Brüel & Kjær (Nærum, Denmark)) according to the DIN 65561 standard and with some deviations from it. Some of these deviations were related to the drop tower build-up and to the drop weight, which was adjusted to minimize the fall distance. The CFRP panel is large, and it was affixed using wood fixtures with pieces of foam under it to protect the structure. Metallic supports were used as well to support the wood structure itself. Some friction might have been caused during the impact and there might have been some energy dissipation. However, the uncertainties are inherent to every impact and are not an issue for the investigation. The acceleration data was recorded using the LTT-184 (128 MB) data acquisition system.

**Figure 8:**
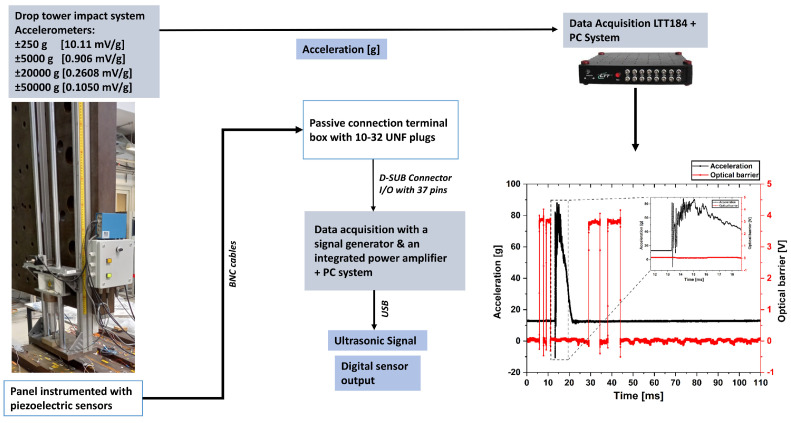
Experimental set-up.

A description of the set-up is shown in Figure 8 and the energies aimed for and experimentally measured, when applicable, are summarized in Table A1.

In Ref. [6], a qualitative classification of specific types of impact damage is introduced, as well as the minimum energy in joules to initiate first failures in a 2.05 mm quasi-isotropic CFRP (PEEK). Based on data from the literature and a step-wise approach to induce damage in a CFRP aircraft structure, several impacts were performed, starting from below 1 joule up to a maximum of 50 joules. Impacts in a range of 1 to 19 joules were used to simulate a tool drop during maintenance work and 20 to 50 joules to simulate runway debris during takeoff or landing and bird strikes at low velocity/energy. All impacts from 1 joule to 45 joules were performed on the inner shell, and one single 50-joule impact was performed on the outer shell of the panel. If the impact is made on the inner shell, damage onset occurs from the outer side of the structure [16]. Since the Lamb waves travel through the whole thickness, conceptually, the difference with an impact induced from the outer shell would only be that in the latter case, the damage propagation would start from the inner side due to deflection. Therefore, in this study, the impacts performed on the inner shell were meant to simulate all types of impacts.

### 4.2. Piezoelectric Sensors

The types of sensors and their physical properties used in this study are presented in Table A2. Each transducer can act as both an actuator and receiver. Pitch-catch and pulse-echo signals were recorded. The actuator was a source of discontinuity. Estimating the time of flight using two signal paths provides a more accurate estimation of the wave velocity. However, in the SHM field, using a pair of sensors is enough for the velocity estimation along the line of sight (one specific direction). First, the effect of the sensor thickness on the signal actuation and reception was experimentally assessed. Sensors with a diameter of 5 mm were fragile. They did not always withstand small impacts and even general handling during gluing. Very special extra care had to be taken during the soldering as well to prevent loosening the negative pole, which is wrapped.

In addition to the data acquisition system, a passive box with 32 channels was used for the external cabling of all sensors. The shear sensors were thickness-poled and allowed the activation of symmetric modes. The disc sensors allow the generation of both modes with a predominance for the activation of anti-symmetric modes.

The Lamb waves are multi-modal, which means that several modes could be simultaneously activated. They are also dispersive, and depending on the thickness and the type of the structure’s lay-up, the signal in the time domain could be very complex to interpret. The signal waveform contains several packets. Every packet represents a mode, a mode conversion, or a reflection indicating an interaction between the wave and a discontinuity or a design detail. Some modes could be canceled out by using strategic sensor positioning. When sensors are placed on both sides of the structure and the actuation is performed in phase, the in-plane symmetric mode could be activated predominantly. If the actuation is perfromed out of phase, the anti-symmetric mode is activated. For aircraft applications, the first configuration is not possible since putting one sensor on the outer shell makes it endure extreme environmental conditions. Therefore, to isolate the symmetric mode and cancel out the anti-symmetric mode, using shear sensors on one single face could be a possible solution. Using an actuation frequency close to the radial resonance frequency of the sensor is a concept that can be investigated for mode discrimination.

In this study, an experimental tuning process with disc sensors is performed to determine the actuation frequency at which the fastest wave mode has the highest amplitude. The investigated structure is divided into five sections, as shown in Figure 6. Each section refers to the space between two stringers. Different types of sensors are glued, most of them with a 10 mm diameter and with varying thicknesses from 0.2 mm to 1 mm. All the piezoelectric wafer active sensors (PWAS) in the two networks are glued using M-Bond AE-10 Epoxy. A small weight is put on the structure after application, and they are left to dry overnight. The list of soft PZT-type sensors used in Networks 1, 2, and 3 is shown in Table A3 and Table A4.

## 5. Damage Detection

### 5.1. Damage Index Definitions and Mathematical Formulations

This section presents the data analytics implemented on ultrasonic data gathered from transducers to gain quantitative conclusions about structural health information.

The technique adopted for damage detection relies on pitch-catch interrogation, where one transducer excites wave propagation and one sensor records the wave traveling through the media. The idea behind this approach consists in the observation of the recorded signal variations according to the waveguide changes in between the sensor pair. A common way to gather information about waveguide modification relies on the use of scalar indicators by comparing the current signal (unknown condition) with a reference signal (baseline or previous inspection) [45].

According to this approach, changes in wave propagation characteristics are correlated to potential structural anomalies. This can be carried out using one or more ultrasonic signal features. However, these signal parameters generally show disparate sensitivities to different types of damage (e.g., crack, delamination, hole, etc.). For this reason, the combination of different metrics can increase the reliability of the damage detection approach, especially when the mechanics of the waveguide is not easy to predict, such as in composite structures. Despite the possibility to detect anomalies in a simple and viable way using the comparative approach, a solid damage index (DI) is pivotal. This requires the investigation of the wave features that return the most effective feedback about the structural condition. From this point of view, the information provided by Lamb waves can be explored using either physics-based or signal-based metrics. In the case of impact-induced damage, the emerging flaw is generally located or distributed through the thickness according to damage severity. This material discontinuity alters the waveguide, affecting Lamb wave dispersion behavior and scattering phenomena (including mode conversion) due to the local interaction between ultrasound and damage [69]. To include any changes arising in the wave propagation, the whole time history can be processed to compute a damage parameter. In this sense, the differential signal energy (E) highlights changes in the current waveform with respect to the baseline signal, providing the damage severity according to Equation (3):

(3) $$DI_{Energy}=\sum_{i=1}^{N_{sp}}{\left(x_{1,i}-x_{2,i}\right)}^{2}$$

where $x_{1}=x_{1}\left(t\right)$ and $x_{2}=x_{2}\left(t\right)$ are the baseline and current digitized signals and $N_{sp}$ is the digital sample number ($x_{1,i}$ is the *ith* sample of $x_{1}\left(t\right)$). Otherwise, the waveguide changes can be identified from wave scattering analyzing transmission and reflection characteristics [70,71]. Among them, the amplitude of the wave traveling across the flaw shows a variation that can be encapsulated in a damage index shape by Equation (4):

(4) $$DI_{SA}=\frac{\left|A_{C}-A_{B}\right|}{A_{B}}$$

where *A* is the amplitude of the most tuned wave mode in pristine ($A_{B}$) and current ($A_{C}$) condition. The indicator obtained from Equation (3) returns the energy content of the differential signal, which is due to the changes in wave propagation. This feature returns a good indication of the damage. However, its value depends upon the energy the transmitted signal delivers and is affected by background noise. Instead, the indicator obtained from Equation (4) returns the amplitude of only a specific wave packet, limiting the noise disturbance. However, it still depends on the delivered energy and often misses much of the signal changes.

Complementary to physics-informed signal processing, a signal-based comparison can be performed between measurements (i.e., current and baseline) resorting to a variety of statistical approaches. The root-mean-square deviation (RMSD) assesses differences among observed (experimentally) and predicted (using an estimator) values. This idea can be extended to find out differences among two samples (or population of samples) that may vary, neither of which is accepted as the “standard”. In this way, a damage index can be defined by computing the average difference between two time series:

(5) $$DI_{RMSD}=\sqrt{\frac{\sum_{i=1}^{N_{sp}}{\left(x_{1,i}-x_{2,i}\right)}^{2}}{N_{sp}}}$$

The same time series can be compared resorting to a correlation index, namely the Pearson correlation coefficient presented in Equation (6), where $\bar{x}$ is the sample set average:

(6) $$CC=\frac{\left|\sum_{i=1}^{N_{sp}}\left(x_{1,i}-{\bar{x}}_{1}\right)\left(x_{2,i}-{\bar{x}}_{2}\right)\right|}{\sqrt{\sum_{i=1}^{N_{sp}}{\left(x_{1,i}-{\bar{x}}_{1}\right)}^{2}{\left(x_{2,i}-{\bar{x}}_{2}\right)}^{2}}}$$

On this basis, a correlation coefficient CC=1 is returned when no mismatch is present in the signals, which ideally happens when no damage occurs between two inspections. Inversely, the correlation coefficient decreases when two signals are not perfectly correlated. This is where damage occurs/increases. Therefore, the correlation coefficient (CC) can be computed in a damage index shape by Equation (7):

(7) $$DI_{CC}=1-CC$$

Despite not including any physics-based information, the indicators obtained from Equations (5) and (7) have the significant advantage of being automatically normalized with respect to the energy content of the incident wave, which inherently reduces noise and background effects.

To look into the damage evolution, the detection indicators mentioned above are used on different concurrent measurements carried out while damage may arise due to impact occurrence according to the procedure discussed in the previous section.

### 5.2. Damage Localization

Using the pitch-catch approach, damage indicators return health information along the line of sight defined by the transducer pair. Combining all possible interrogation paths identified by a network of transducers, the damage can be localized, too. The data processing strongly depends upon the reconstruction algorithm used to combine the information collected along all possible paths. Single data need to be weighted and transferred to the structure mesh where the damage probabilistic distribution is estimated in each specific node (tomographic representation).

**Figure 9:**
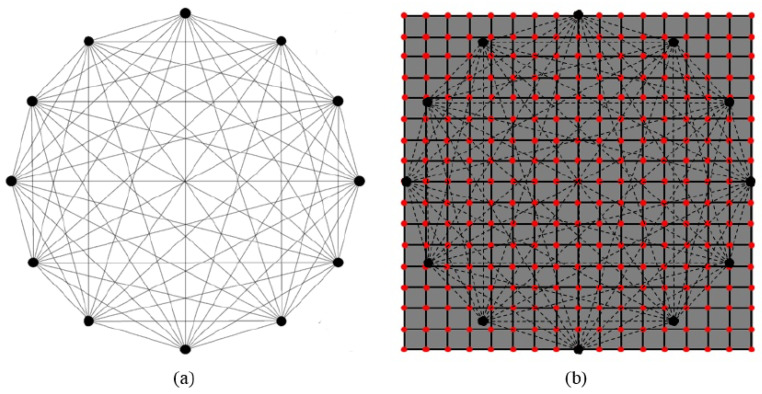
Piezoelectric transducer array and resulting propagation paths where damage indices are calculated (**a**). Structural mesh where damage probability index is derived using probability distribution function (**b**) [74].

Among various techniques, Lamb wave data are usually processed using probability-based diagnostic imaging because it is compliant with the few resources available [72,73]. Using the pitch-catch approach for all transducers actuated individually, the SHM inspection returns the network shown in Figure 9a. Repeating the interrogation multiple times, damage indicators are obtained every time an inspection is carried out.

The path-related information gathered by the DI is then distributed all through the structure resorting to a weight distribution function based on the definition of a distance index (dI). The distance index introduces a probability for every node of the mesh, which decreases with the distance of the node from the path. The combination of damage and distance indicators finally returns a damage probability index (DPI) for every point of the mesh as shown in Figure 9b. The *i*th path interrogation contributes to the probability index in point P as follows:

(8) $$DPI_{i}\left(P\right)=DI_{i}\cdot dI_{i}\left(P\right)$$

where $dI_{i}$ introduces the weight of the distance between the *i*th path and P, resulting in a decreasing probability. Finally, the DPI is computed in any point P normalizing the cumulative effect in P of every possible path:

(9) $$DPI\left(P\right)=\frac{\sum_{i=1}^{n}DPI_{i}\left(P\right)}{max(DPI)}$$

Regarding the weighting distribution function, distance indices are computed using different approaches. In the first approach, the actual distance δ(P) value is calculated as the norm between point P and the line of sight identified by the transducer pair. The second approach returns a distance on the ellipse characterized by the transducers as focal points. As a result, the iso-probability lines are elliptical.

Finally, to limit the analysis to the area surrounding the transducer, the first approach is modified considering the minimum distance among the norm between the point and the line of sight and the norm between the point and the transducers. The distance index is then evaluated following Equation (10).

(10) $$dI=\left\{\begin{array}{c} \frac{\beta -\delta (P)}{\beta -1},\;if\;\delta \left(P\right)\leq \beta \\ \;0\;,\;if\;\delta (P)\geq \beta \end{array}\right.$$

where δ(P) is the distance mentioned above and β identifies the maximum distance of influence. Indeed, the DPI suddenly vanishes when the distance is greater than β, as the path does not influence that point. The definition of the β value is not trivial and it is calibrated from a known condition to make the reconstruction more reliable [74]. Otherwise, several approaches have been proposed in the literature to optimize it [75,76].

## 6. Results

### 6.1. Theoretical Calculations of the Dispersion Curves

**Figure 10:**
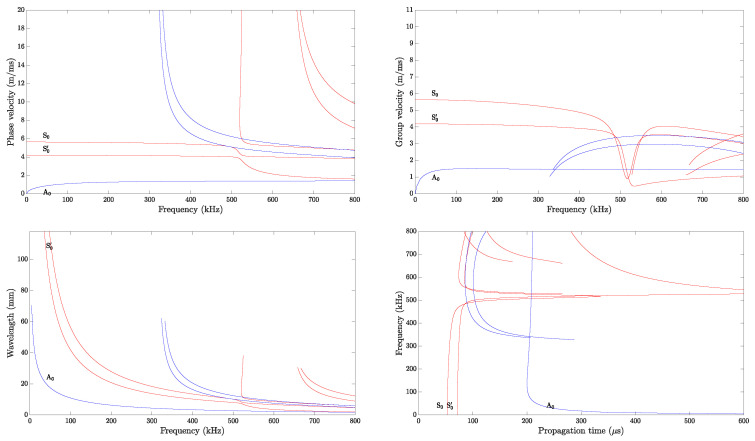
Dispersion curves for the propagation direction 0° displaying first modes: S0 (symmetric), S0’ (shear) and A0 (anti-symmetric).

**Figure 11:**
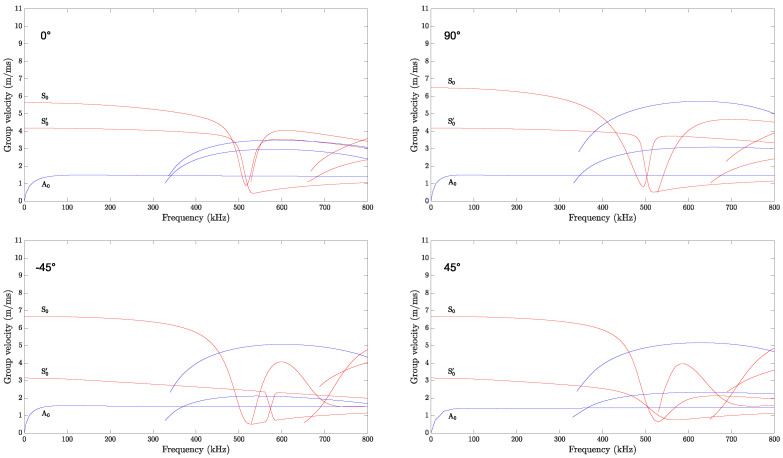
Group velocities in all four propagation directions.

The dispersion curves (i.e., frequency versus phase velocity, group velocity, propagation time, and wavelength) are generated considering an infinite plate having a lay-up and similar material properties to the investigated structure (i.e., a realistic CFRP panel). The excited modes in a structure depend on the material and frequency thickness. The Dispersion Calculator software v1.10, which relies on an analytical solutions system by applying the stiffness matrix method [77], is used. The dispersion curves for the 0° propagation direction, computed for a propagation distance of 300 mm, and the group velocities for all four propagation directions are shown, respectively, in Figure 10 and Figure 11.

The wave velocity values obtained from the simulation results, calculated analytically, are higher than those determined experimentally. A similar trend can be observed in the work of [78]. For the phase and group velocity dispersion curves, it can be seen that in the frequency range of 0 to 300 kHz, only three fundamental wave modes coexist: the anti-symmetric (A0), the symmetric (S0), and the shear mode (S0’). If the actuation occurs above that range, higher modes appear, and the signal waveform contains further wave packets. Examples of the signal waveform at different actuation frequencies are shown in Section 6.3.

### 6.2. Experimental Tuning Process and Sensor Dimensions

**Figure 12:**
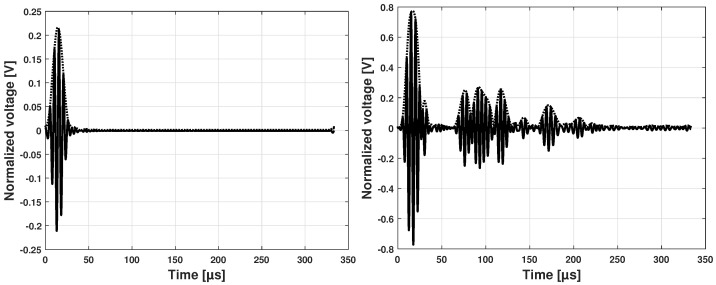
A typical 5-count burst actuation waveform at 190 kHz (**left**) and a receiver signal (**right**).

After the frequency range with the less dispersive behavior was determined using the theoretical dispersion curves, an experimental tuning process was performed. The tuning process consisted in experimentally determining the maximum peak amplitude of the arrival wave for some wave paths. The determination of this value has to be performed for the same test configuration since the amplitude of the receiver is strongly dependent on the actuation amplitude and circuitry gain applied. An example of the burst actuation and the received signal is shown in Figure 12. Both Sensors 1 and 2 in Section 3 have a thickness of 0.5 mm and are aligned along the 0° propagation direction.

**Figure 13:**
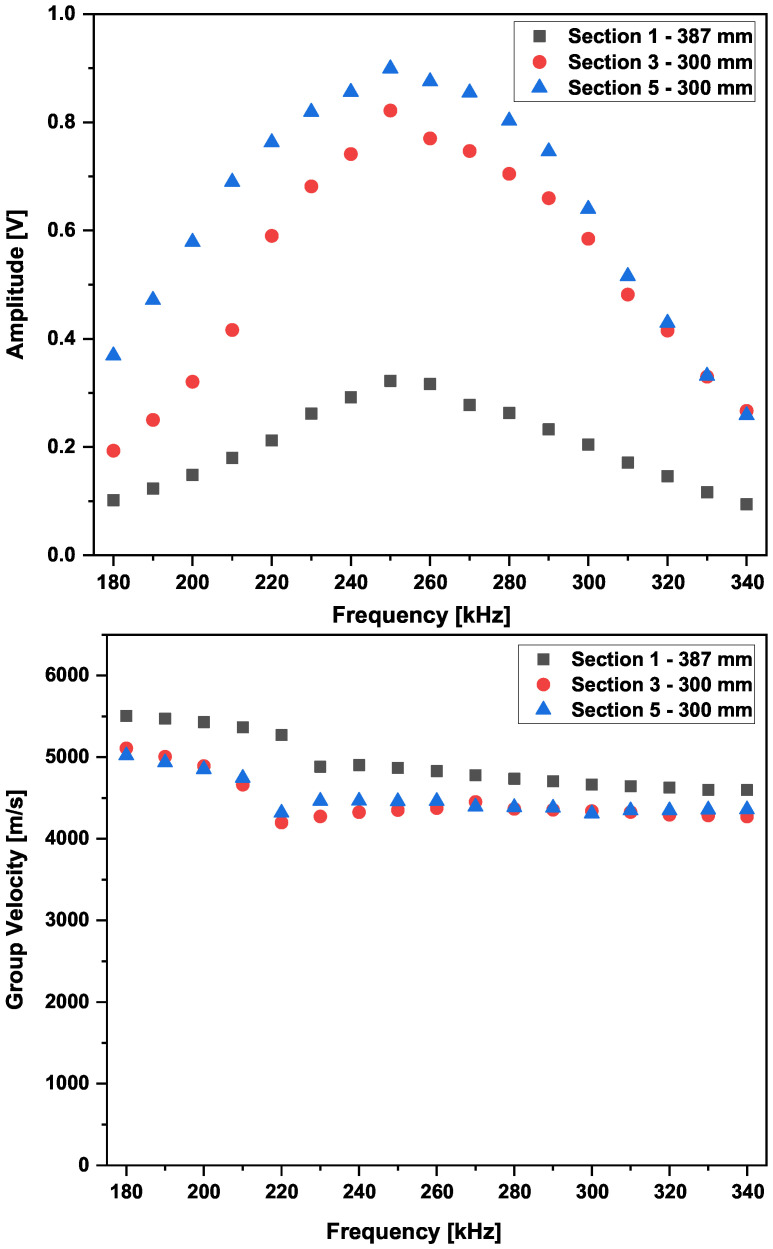
Experimental tuning process: (**top**) peak voltages over a frequency range, (**bottom**) group velocities in three sections of the panel.

For three particular wave paths in Sections 1, 3, and 5, the amplitude of the first arrival waveform of the S0 mode was recorded, and the results are shown in Figure 13.

**Figure 14:**
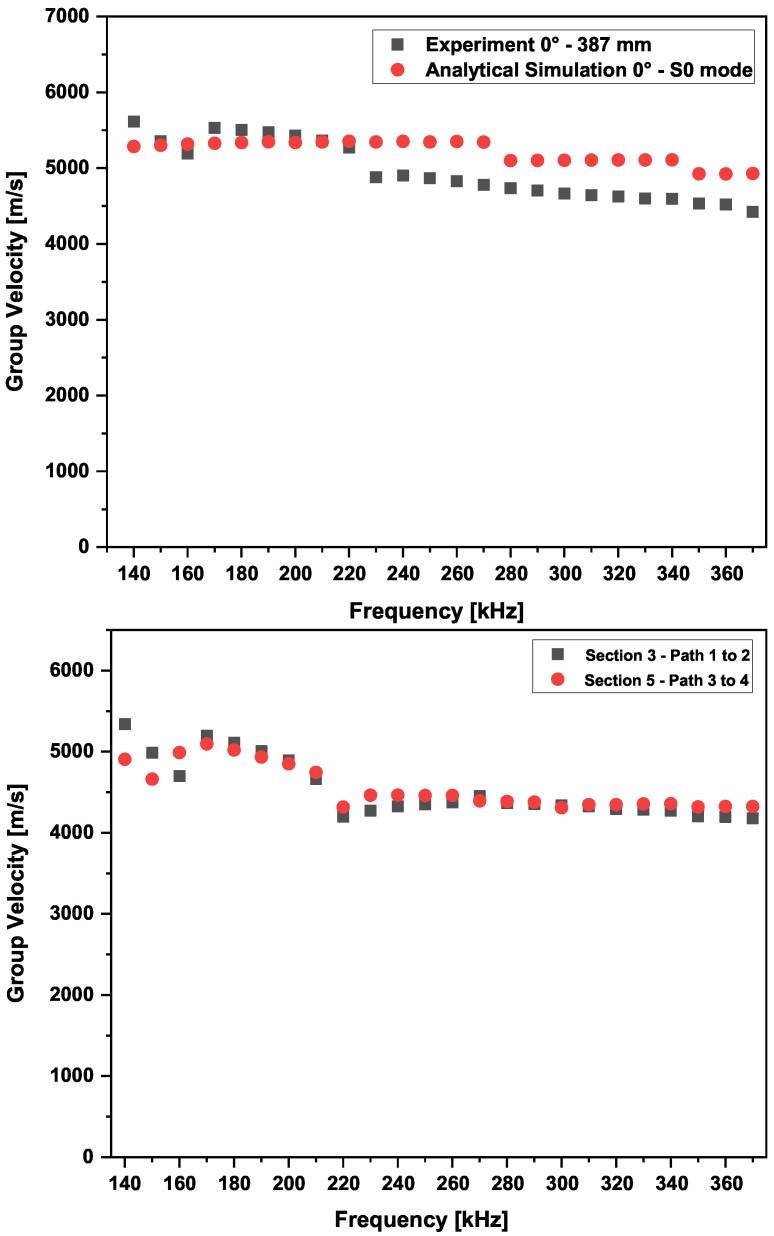
Group velocity: (**top**) Experimental versus analytical wave velocities for wave Path 2 to 14, (**bottom**) group velocity measurements in Sections 3 and 5.

The results of the experimental tuning process show a peak at 250 kHz. Therefore, as a first step, the group velocity obtained at this frequency value was used for the damage detection process. The wave velocities of the first wave packet were calculated using the peak of the Hilbert transform, and a comparison with the theoretical analytical values is presented in Figure 14.

The results show that the wave velocities obtained from the simulations based on the stiffness matrix method [79] were mostly higher in value than the values obtained from the experiments. Indeed, this is inherently due to the transient phenomena involved in wave propagation, which can only be captured through experiments. The discrepancy arises from how the velocity is calculated between the actuator and receiver, which is affected by transient excitation and aliasing. This leads to a systematic error that depends on the distance between the transducers [80].

Experimentally, the best waveform is obtained at a center frequency range of 220 to 270 kHz, provided a reasonable distance between sender and receiver for the applied actuation voltage is achieved.

For the damage detection process, a five-count burst actuation pulse was selected. In Ref. [81], the effects of minimizing geometric effects through the design of a piezoelectric disc and the concept of the minimum resolvable distance (MRD) were discussed.

The sensor’s thicknesses and actuation signals effects are assessed in all five sections. Different types of piezoelectric sensors were glued and thoroughly analyzed. The type of wave received depends on different factors: the structure thickness and material properties, the sensor properties, the bonding quality, and the data acquisition system used.

To assess the effect of the PZT thickness on the signal transmission quality, pulse-echo signals were recorded along the pitch-catch waveforms. It was noticed that the lower the actuation frequency, the less distorted the actuation pulses. The optimum actuation frequency was determined through the experimental tuning process explained above.

Sensor discs with a thickness of up to 1 mm are still suitable. However, the smaller the diameter (i.e., 5 mm and 6.5 mm), the less resistant they are to the aftermath of an impact and during the gluing and soldering processes.

In conclusion, to obtain an optimal waveform, a sensor of diameter above 5 mm and a thickness below 1 mm was experimentally proved to be suitable. For the shear sensors, the opposite was noticed; the thicker the sensor, the better the actuation and the reception of the ultrasonic waves. A 3 mm thick shear quadratic sensor provided better results than a 0.5 mm thick sensor in terms of the actuation and reception of waves. The results are structure-dependent.

### 6.3. Experimental Wave Reception in a Multilayered Structure

**Figure 15:**
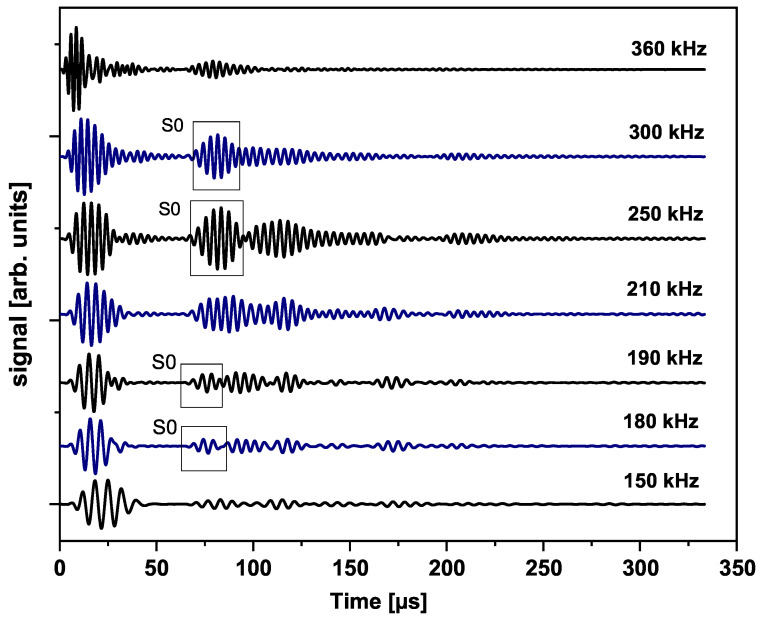
Received signals as a function of frequency.

**Figure 16:**
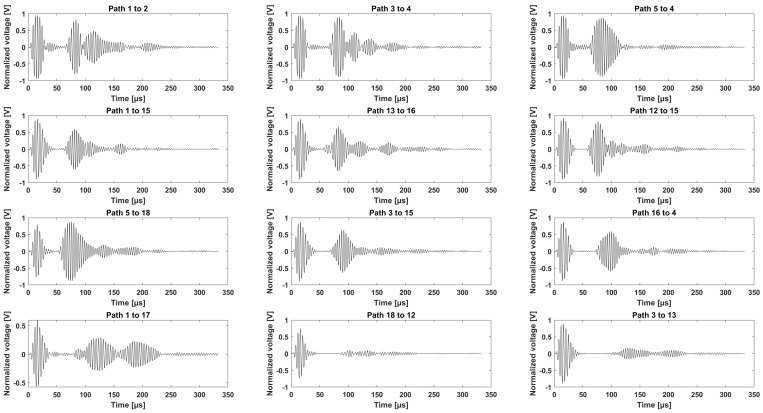
Waveforms in network no. 3: In Sections 3 and 5, crossing Sections 3 to 4, 4 to 5, and 3 to 5.

The fundamental symmetric mode was experimentally generated using wraparound electrode pattern-type sensors: circular-shaped sensors and one shear sensor. The panel was subdivided into five different sections. For Sections 3 to 5, a subnetwork of six PZT sensors, positioned in the propagation directions of 0°, ±45°, and 90°, was investigated. The received signals as a function of frequency for a sender–receiver separation distance of 300mm±1mm are depicted in Figure 15. The different propagation routes, in Sections 3 to 5 as described in Figure 4, are shown in Figure 16.

The amplitude of the S0 mode reaches a maximum at a frequency of 250 kHz. The A0 mode cannot be distinctively located. In the frequency range between 140 kHz and 360 kHz, it is still possible to record a good signal quality where the S0 mode waveform can be localized. From 360 kHz, the wave packets are very compact below 100μs, and the determination of the first wave packet becomes very difficult. Below 90 kHz the wave can barely be detected, and a well-formed wave packet cannot be seen. For this 2.36 mm thick structure, more sensitivity for sensing is observed for frequencies above 100 kHz and below 400 kHz (i.e., the excited modes have larger displacement at the plate surface).

### 6.4. Lamb Wave Signatures in an Aircraft Primary Structure with Stiffeners

This section presents Lamb wave signals measured in the stiffened fuselage segment at the center actuation frequency of 250 kHz used for damage detection.

**Figure 17:**
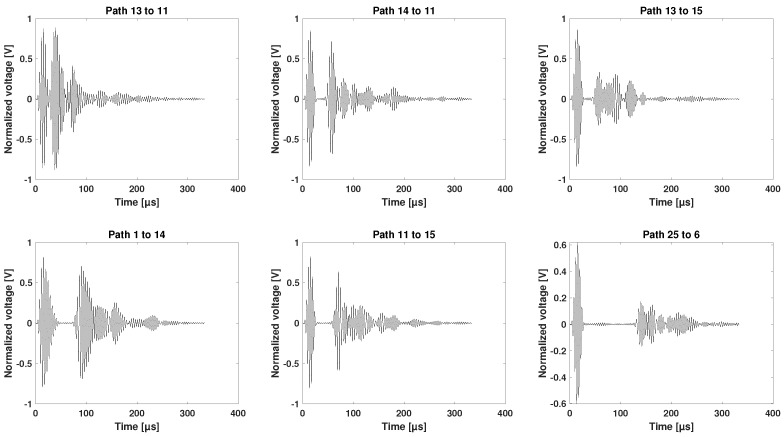
Waveforms in Section 1.

The experimental data provide an estimation of the group velocity. In Figure 17, a selection of waveform signatures for different paths in Section 1 is shown. Four hundred and twenty-one paths were recorded using 25 sensors.

The wave paths displayed in Figure 17 are along the stringers, which creates side reflections. Nevertheless, the first wave packet can be well distinguished for short propagation routes, such as Path 13 to 11 (124 mm), and for longer propagation routes, such as Path 25 to 6 (674 mm).

A selection of signals in the time domain, collected from the newly designed network in Sections 3 to 5 before the 50 J impact on the outer shell, are shown in Figure 16.

Following the first level study of damage detection in Sections 3 to 5, Sensors 11, 12, 13, 14, 15, and 16 were added to the sensor layout of Network 2 (see Figure 3) to generate Network 3 (see Figure 4) and improve detection performance. Two hundred and ninety-two paths were recorded using 25 sensors.

In Figure 16, a small selection of signals from the new network is displayed. The first row contains signals recorded using Sensors 1 to 5 (Path 1 to 2 and Path 3 to 4 in the 0° direction and Path 5 to 4 in the −45° direction). The next three successive rows consist of the following signals: wave paths between Sections 3 and 4, wave paths between Sections 4 and 5, and wave paths across two stringers from Sections 3 to 5.

Both Figure 16 and Figure 17 show the variety of the wave paths and the capability of the designed experiment in measuring adequate signals from various sender–receiver distances at different angles and propagating in-between, across, and along the stiffeners of the CFRP panel.

### 6.5. Measurement of Group Velocity

The experimental wave path data allow the determination of the group velocity and the onset time of arrival of the different wave modes. In the recorded signal measurements in this study, the second wave packets correspond to the symmetric mode S0.

**Figure 18:**
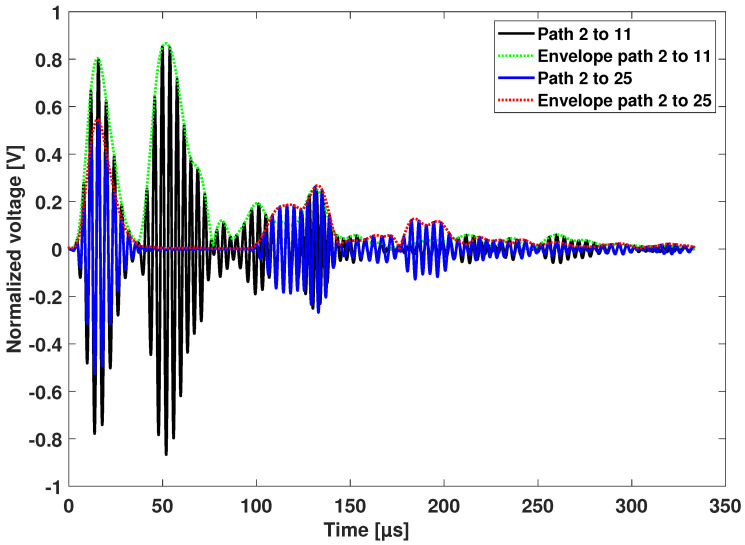
Wave Paths 2 to 11 and 2 to 25.

One actuator and two sensors are used to have a precise wave velocity estimation and a precise time of flight (i.e., time of flight from sender to receiver). The determination of the onset time of a particular wave mode is achieved using the peak of the Hilbert transform. The data shown in Figure 18 were collected before the 20-joule impact and at a center actuation frequency of 250 kHz.

The group velocity is determined by measuring the arrival times at the respective sensors from one sender, in this case sensor number 2 in Section 1. The wave velocity is equal to the Euclidean distance between Sensors 11 and 25 divided by the time difference between the two Hilbert peaks of the two arrival waves. In this case, the actuation time is not required.

The obtained group velocity was found to be equal to 5288.01 m/s. When relying on the two sensor receivers, the effects of the source are not present. The obtained group velocity is higher than when using one actuator and one sensor only. If only a pair of sensors were used, the group velocity would be equal to 4654.88 m/s and 5061.85 m/s for Path 2–11 and Path 2–25, respectively.

### 6.6. The Experimental Reference Design Model

The panel as depicted in the experimental section was composed of six stiffeners. A network was built to assess wave propagation following different routes. Section 5 was kept intact, in a pristine state, during the entire test campaign. No impacts were performed on that part. A sensor configuration composed of two pairs of sensors, separated by a distance of 300mm±1mm, was placed in Sections 3 and 5. Since no robotic system placed the sensors, minor differences could be assumed in the sensor application and positions. The wave propagating between these sensors in the two sections was compared as part of a reference design model.

**Figure 19:**
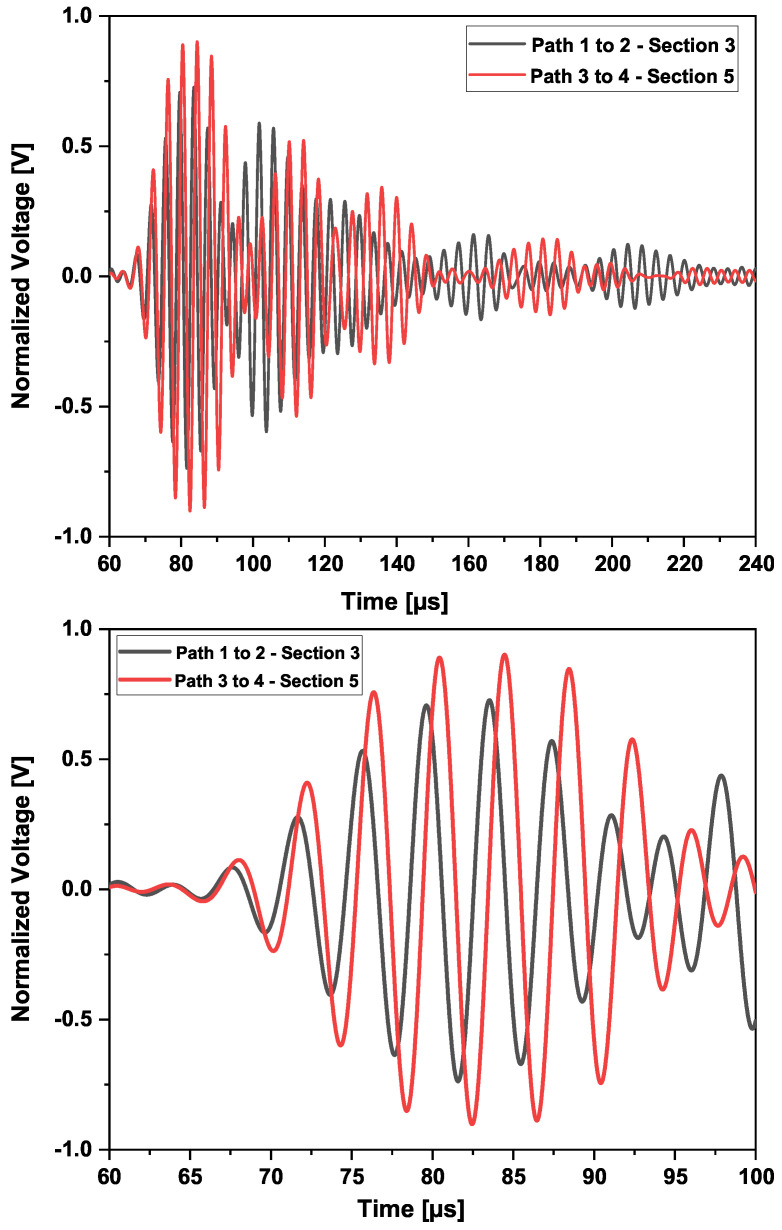
Waveforms in Sections 3 and 5 at a center frequency of 250 kHz: (**top**) experimental received signal, (**bottom**) first direct wave.

In the middle of the path of the sensors placed in Section 3, impacts with energies between 5 joules and 45 joules were performed. Every time a comparison was performed, it was not only with the pristine state of Section 3 but also with the permanent pristine state of Section 5. The waveforms before the series of impacts of 7, 30, and 40 joules are shown in Figure 19.

**Figure 20:**
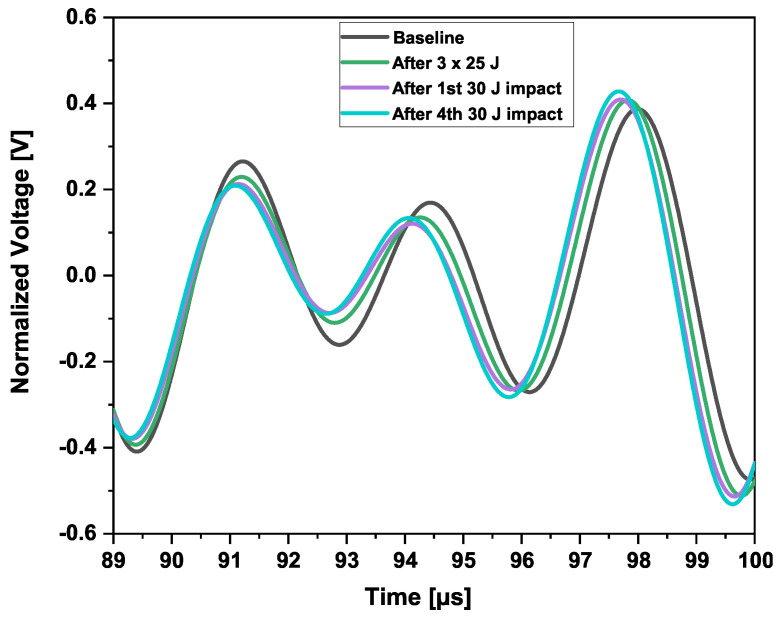
Effects on waves’ phase and amplitude.

A phase shift was observed between the two waveforms of the two sections. This is mainly due to differences in the sensors’ application. The distance center to center aimed between Sensors 1–2 in Section 3 and Sensors 3–4 in Section 5 was 300 mm. After application, the center-to-center distances were about 300mm±1mm. This difference caused an 8μs phase shift, which should be taken into consideration. However, a small difference in amplitude could be observed as well. The presence of damage does not immediately mean that the wave velocity is reduced. As can be seen from Figure 20, the baseline waveform is the slowest after the first arrival wave mode. In addition, the further away the sensor is from the actuator, the faster the wave could become.

**Figure 21:**
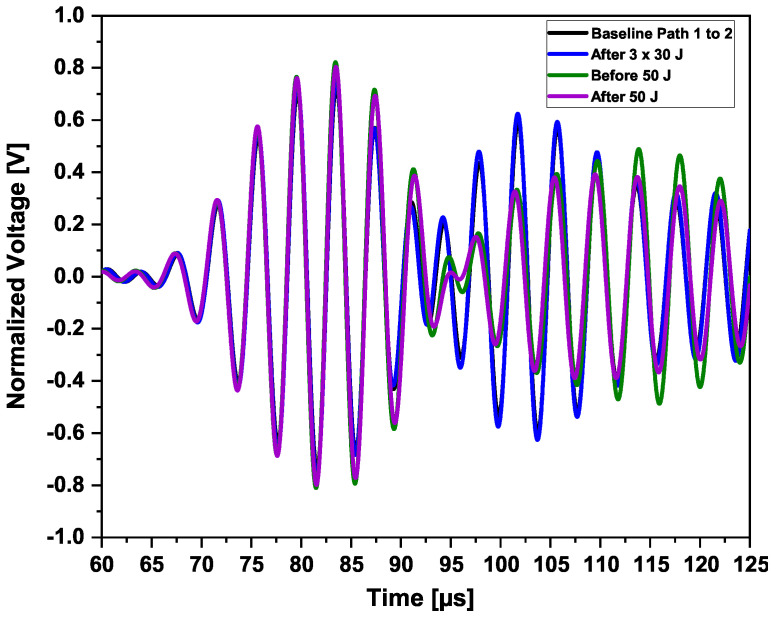
Waveforms after a series of impacts: The first wave packet.

One single impact of 50 joules was performed on the outer shell of the CFRP structure, at the same locations where all impacts on the inner shell in Section 3 were performed. Comparisons were performed between the signal waveforms, before, and after impacts, in Section 3. This is the first analysis in the time domain before the image reconstructions, as displayed in Figure 20 and Figure 21.

### 6.7. Angular Dependency of the Wave Velocities

The investigation was performed in Sections 3 to 5. The ±45° angles were realized by placing sensors on both sides of a stringer to realize the minimum distance required to obtain a good waveform. The angles are depicted in Figure 4.

**Figure 22:**
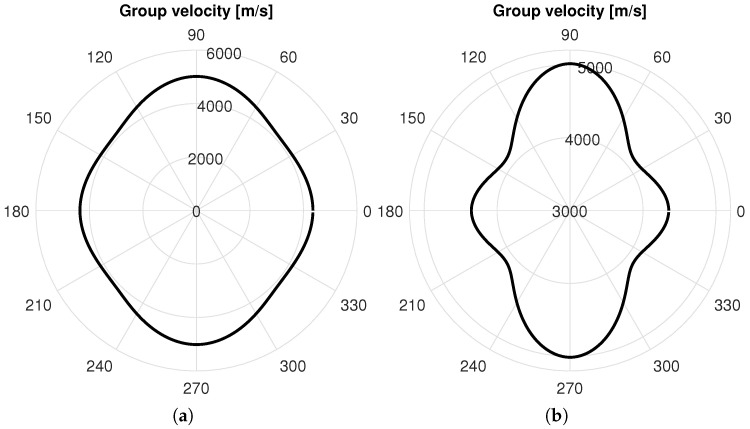
The polar pattern of experimental group velocity at 250 kHz. The nominal scale (**a**) and enlarged scale (**b**) used to highlight the variability over the propagation direction.

Figure 22 shows the polar pattern of experimental group velocity at 250 kHz. The nominal scale in Figure 22a shows that the wave profile slightly varies with the direction. This change is better represented in Figure 22b, enlarging the radial scale, which allows the variability over the propagation direction to be highlighted.

The group velocity was calculated along ±45°, 0°, and 90° using the time of flight. Then, the velocity along other directions over the pattern was extrapolated using constrained spline interpolation. As to the wave propagation, according to the direction, the wave either travels directly to the sensor or encounters some discontinuities due to the stringers.

In this latter case, the waveform is strongly affected but still good to evaluate the wave propagation and the time of flight qualitatively. Although the angular dependency of the wave velocity is present, the variability over the pattern is not high due to the limited level of laminate anisotropy. This is mostly due to the thickness of the plate and the high number of plies oriented in different directions, providing quasi-isotropy to the waveguide. Nonetheless, the use of model-based approaches is strongly complicated by this aspect, and it is rather worth relying on data-driven approaches.

### 6.8. Tomographic Image Reconstruction

A probabilistic imaging approach consisting of the determination of the probability of having damage according to a weighting distribution function is used for the damage detection and localization process. The number of actuator–sensor paths varies between 52 and 421 paths and the center frequency used is equal to 250 kHz, as determined in the tuning process.

A three-stage approach is used. First, the detection area is restricted to Section 1, then Sections 1 to 2 when one stringer is crossed, and then Sections 3 to 5 when two stringers are crossed. In the latter case, reflections could come from three stringers.

The maximum number of paths considered for Sections 1 to 2 and Sections 3 to 5 are 421 and 292 paths, respectively. In both cases, the maximum number of sensors is equal to 25.

Damage indicators based on the amplitude and time of flight are not considered when a high number of paths (e.g., 400) is used.

**Figure 23:**
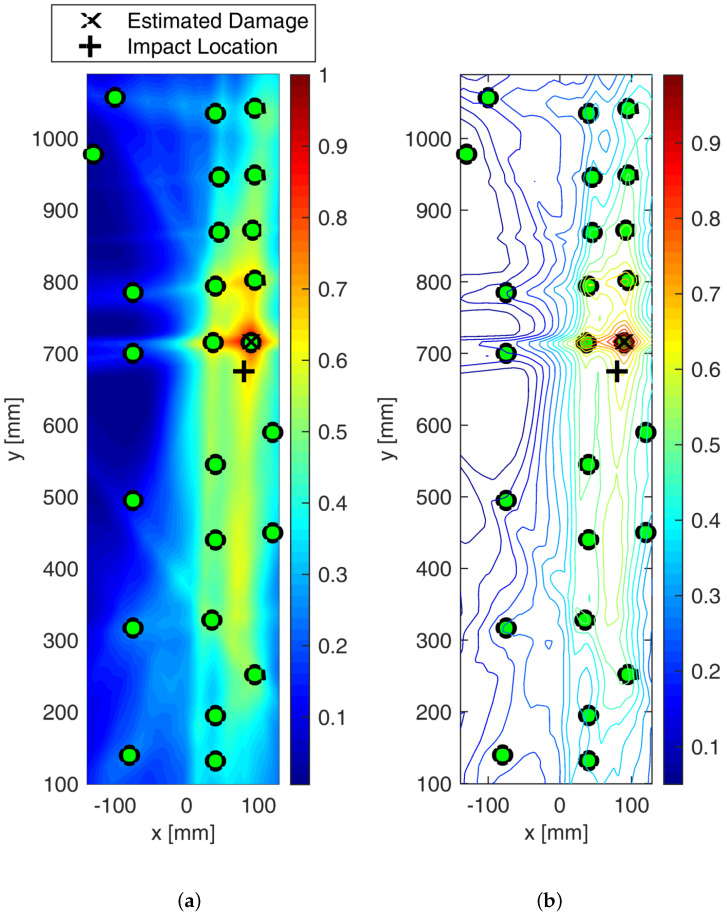
Damage probability index maps relying on an energy-based index. The damage map (**a**) and contour plot (**b**) are obtained using the linear weight distribution function. Colorbar in the range [0–1]. SHM carried out at 250 kHz.

Figure 23 shows the detection of impact-induced damage on Section 1 resorting to the extended sensor network located in Section 1 and Section 2. The reference inspection is carried out at 250 kHz and the diagnostic signal is a 5-sine cycle windowed by the Hanning function. The current inspection was carried out in the same way after Section 1 was impacted by up to 20 joules. The digitized time histories were then post-processed to detect and localize the damage as extensively described above. Figure 23a shows the colored image plot of the damage probability index, hereinafter called the damage map. This damage map is first normalized in the range of [0–1] and then distributed over the color scale.

The contour plot showing the iso-probability curves is represented in Figure 23b. The reconstruction was achieved through single- and multi-parameter inspection adopting the damage indicators described above. Figure 23 shows the SHM diagnosis using a single-feature analysis based on the energy of the differential signal and a linear weight distribution function. The damage map is quite smooth, revealing the highest probability of damage very close to Sensor 1. The error in localizing the impact position is quite negligible and mostly due to the low sensor density around it. Although the localization was successfully achieved, the imaging is not as sharp as expected when moving away from the impact site. This is due to some artifacts appearing far from the impacted area (not negligible damage probability indexes). This is mostly due to the low level of changes in the signals all through the plate combined with the very tight sensor configuration in section one. As a result, a small variation recorded in wave propagation far from the impact (e.g., either caused by noise or due to environmental disturbances) can also return an imaging artifact. This issue is well known from the literature, especially when a very noisy index like the energy-based feature is used. As shown in Figure 23b, this results in very large iso-probability regions in the range [0.5–0.7].

**Figure 24:**
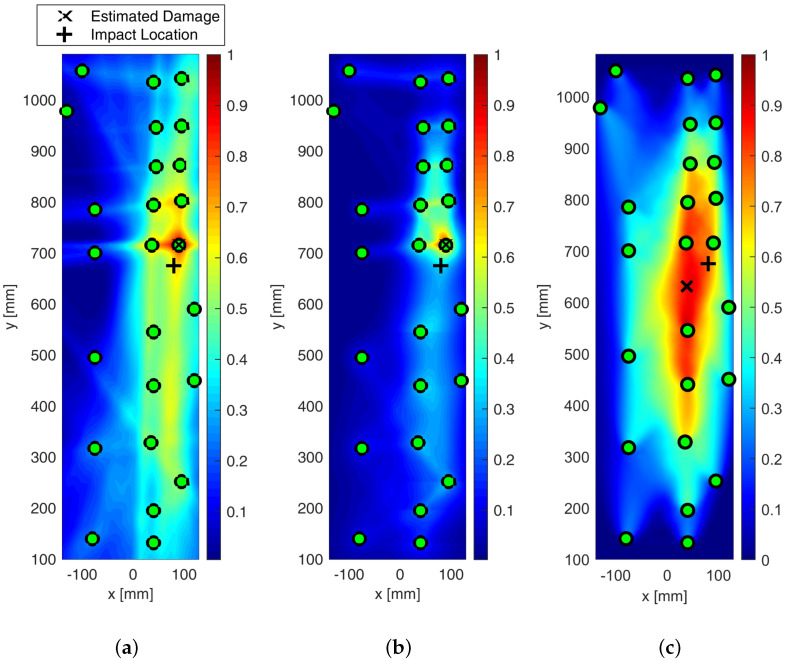
Damage probability index maps relying on energy (**a**), RMSD (**b**), and correlation coefficient (**c**) indices. Colorbar in the range [0–1]. SHM carried out at 250 kHz.

The effect of metrics adopted to build the imaging up is shown in Figure 24. The probabilistic reconstruction all through the structure was performed by employing different distribution weighting functions and optimizing the β parameter.

However, in every case, the localization was achieved with negligible error. In particular, the statistical analysis based on RMSD, reported in Figure 24b, returned very sharp imaging with still a peak of damage probability around Sensor 1. Indeed, moving away from the impacted area, DPI decreases rapidly. Nonetheless, a DPI close to 0.5 is strongly visible in the northern area of Section 1. This is inherently due to the network composition, which returns a non-homogeneous path density through the whole structure. However, this effect does not prejudice the reconstruction. Finally, a slightly different discussion arises around the cross-correlation-based diagnosis depicted in Figure 24c. In this latter case, however, the area with the highest DPI is quite wide, generating a big area of damage iso-probability. Nonetheless, the imaging still looks very well shaped, with the damage map being smoothly distributed without any artifact and the impacted area still well detected and localized.

**Figure 25:**
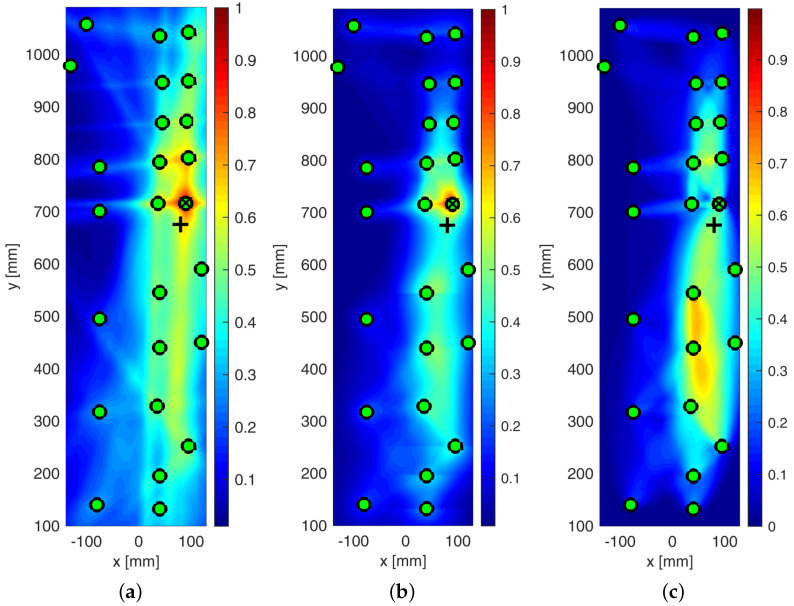
Damage probability index maps relying on a multi-parameter approach. Damage map and contour plot are obtained using linear (**a**), modified linear (**b**), and elliptical (**c**) weight distribution functions. Colorbar in the range [0–1]. SHM carried out at 250 kHz.

The effect of the weighting function is shown in Figure 25, which reports the maps of damage using multi-parameter diagnosis while varying distribution action over the structure.

Although the localization of impact is well achieved, it is evident from Figure 25b how the linear distribution modified to hoop the reconstruction around the transducers shows the most promising localization. Although localization is always achieved in a satisfactory way, the modified-linear weight function usually returns the sharpest reconstruction possible. Instead, the classic elliptical reconstruction likely returns multiple artifacts, as shown in Figure 25c.

**Figure 26:**
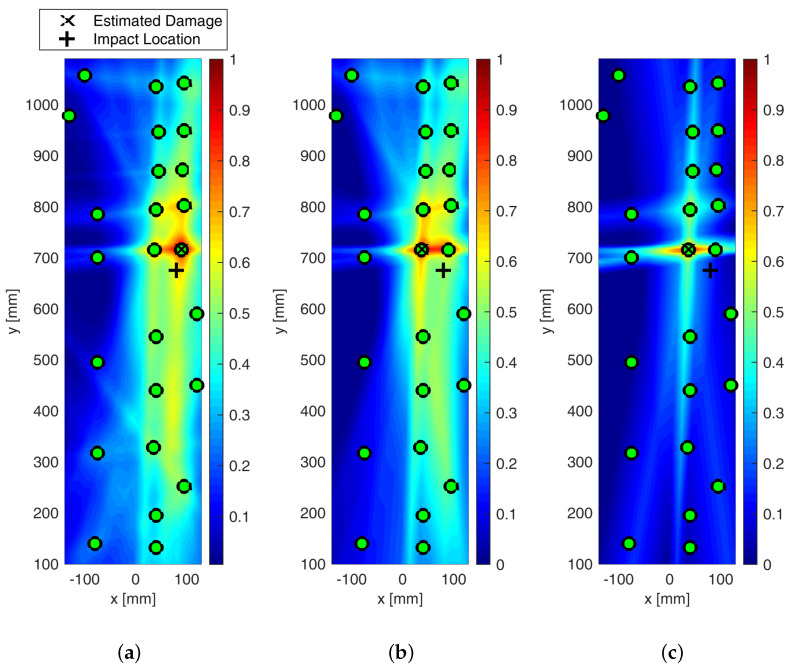
Damage probability index maps relying on an energy-based index. Damage map and contour plot are obtained by applying 0% (**a**), 30% (**b**), and 50% (**c**) thresholding. Colorbar in the range [0–1]. SHM carried out at 250 kHz.

All the results depicted above were obtained either using single features extracted from the raw signals or a combination thereof. However, an important issue to tackle deals with the decision framework, namely setting the level of data that returns useful information about the damage. That is to say, it is important to define a threshold to discriminate damage information from noise. Without looking into further details about threshold definition, which is mostly a matter of weighting uncertainty and knowledge, the effect of its introduction on the damage map is reported in Figure 26.

From the pictures, it is evident how the threshold impacts the imaging and even the damage localization. However, it is of common understanding that increasing the threshold makes the image sharper, increasingly confining the damaged area. In addition, it is worth noting that, in this case, increasing the threshold does not alter the localization so much. This trend usually results when damage is correctly detected and localized, and it is not affected by uncertainty at all.

Section 1 and Section 2 include groups of sensors of the same diameter but different thicknesses, as described in Table A3. The dimensions of the discs are as follows: (10 mm × 0.35 mm) and (10 mm × 0.5 mm), (10 mm × 0.2 mm) and (10 mm × 1 mm). The variations in thicknesses were used to experimentally assess the adequate sensor dimension and also to assess wave propagation through and in between the stringers. The inner shell of the structure has thickness variations. After basic imaging resorting and manipulating all available data at a single frequency, the damage detection is performed in the following cases to rule out the roles played by the sensors’ dimensional similarities and the intensity of the sensors’ coverage.

- With all sensors in Section 1, without sensors in Section 2. In this case, the crossing of the stringers that adds to the complexity of the analysis is reduced.
- With sensors of the same dimensions only.
- With alternate rays of sensors.

The damage detection is performed at different stages of the impact series: after 7 joules, after 10 joules, and after the series of 20 joules energy impacts.

**Figure 27:**
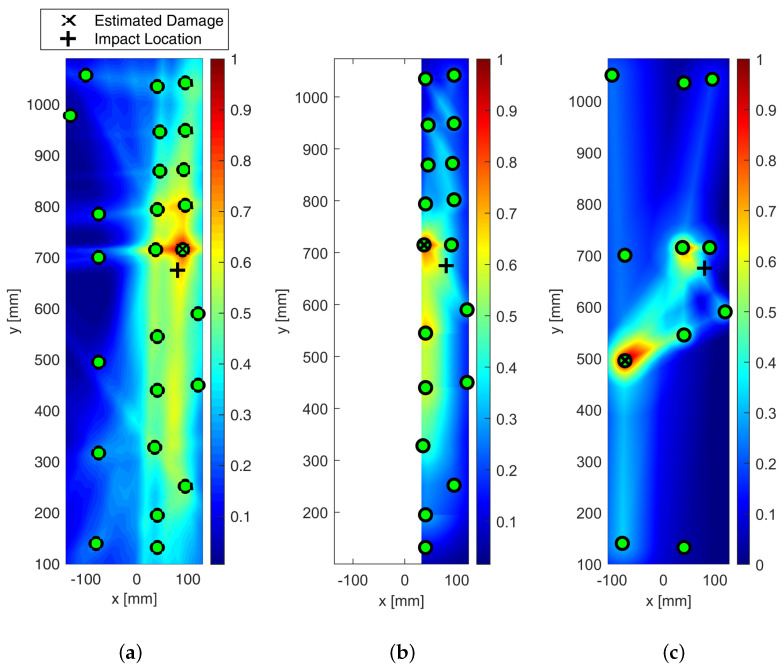
Damage probability index maps relying on an energy-based index. Damage map and contour plot are obtained using 421 paths (**a**), transducers from Section 1 (**b**), and 55 paths (**c**). Colorbar in the range [0–1]. SHM carried out at 250 kHz.

Moving into the details, Figure 27 shows the effect of reducing the number of transducers adopted and, as such, the number of inspection paths. First of all, Figure 27b depicts the diagnosis obtained using the energy-index damage indicator when resorting to the transducer located in Section 1 only.

Although the distribution of DPI is changed due to the reduced number of paths adopted to interrogate the structure, detection and localization of the impact are still possible. As shown by the imaging, the reconstruction is not the sharpest possible. Nonetheless, this result is of high importance due to the complexity of the network. Indeed, the transducers are very close to the stringers, whose boundaries scatter the wave propagation strongly and complicate the analysis. A further insight into the transducer-dependent imaging is given in Figure 27c, where a limited number of transducers is adopted to reconstruct the damage (less than 40 out of 55 available are adopted). In this case, the limited amount of data does not suffice to achieve correct damage localization.

To have a better look into the sensitivity of the damage reconstruction approach while using a limited number of resources, a similar investigation is repeated resorting to the same transducers, but employing different damage indicators.

**Figure 28:**
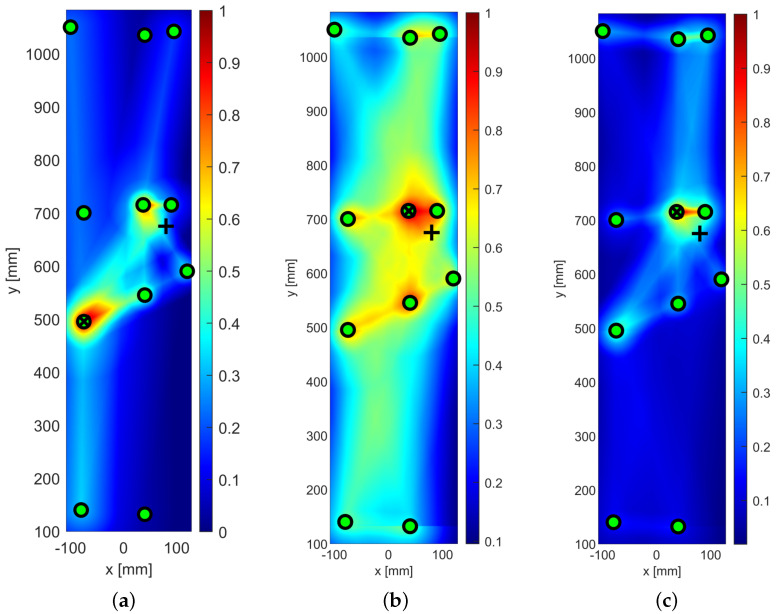
Damage probability index maps relying on a few transducers (55 paths). Damage map and contour plot are obtained using energy (**a**), cross-correlation (**b**), and RMSD (**c**) indices. Colorbar in the range [0–1]. SHM carried out at 250 kHz.

In particular, Figure 28 compares the results obtained with an energy-based indicator (a) with those from cross-correlation coefficient (b) and RMSD (c) analyses, respectively. While the former fails to localize the impacted area, the cross-correlation analysis provides a better visualization of the actual state of the structure, with a hotspot visible very close to the impact location. However, the imaging is not sharp enough to suggest reliable information about the damage. In contrast, this is the case with the RMSD-based index, which is well suited to visualizing the impact area despite the limited amount of data available.

**Figure 29:**
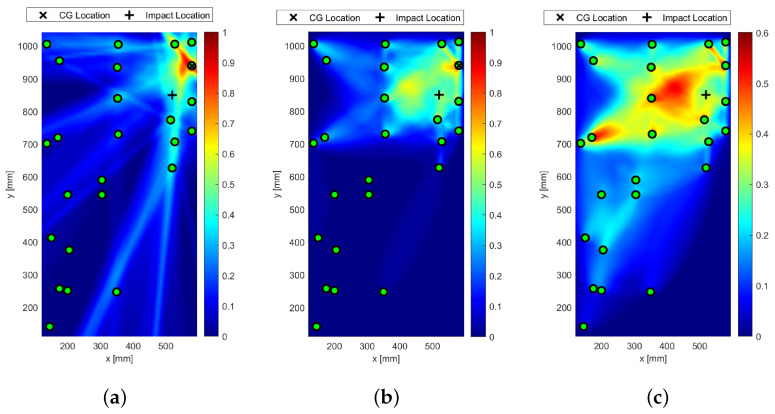
Damage probability index maps in Section 3 using a populated sensor array. Damage maps are obtained using energy (**a**), RMSD (**b**), and multi-parameter (**c**) indices. Colorbar in the range [0–1]. SHM carried out at 250 kHz.

A final investigation was carried out to detect an impact in Section 3. Figure 29 shows the detection of impact-induced damage, resorting to an extended sensor network located all through Section 3, Section 4, and Section 5.

The reference inspection is carried out at 250 kHz, and the diagnostic signal is a five-sine cycle windowed by the Hanning function. The current inspection is carried out in the same way after Section 3 is impacted by up to 50 joules. The digitized time histories are then post-processed to detect and localize the damage as extensively described above. The DPI is first normalized in the range [0–1] and then distributed over the color scale. Figure 29a shows the damage map achieved by a single inspection adopting the energy-based damage indicator. The identification can be correctly achieved even though the localization is not fully accurate. Indeed, the hotspot is slightly shifted compared to the impact location. However, the distance is still rather limited. Likewise, Figure 29b shows the damage map achieved by a single inspection adopting the RMSD-based damage indicator. Again, the damaged area is correctly identified and localized. However, the hotspot is less evident and tiny compared to the energy-based investigation. Finally, Figure 29c shows the damage map achieved with the multi-parameter inspection. There is still a correct identification of damage with a hotspot area concentrated around the impacted area. However, the presence of different hotspots, as well as some artifacts and the lack of the estimation of the center of gravity (CG) location, makes the unambiguous localization of the damage not possible. Indeed, the center of gravity (CG) cannot be calculated within the multi-parameter analysis because of the normalization and fusion of data performed to combine different parameters.

### 6.9. Classical Ultrasonic Testing

**Figure 30:**
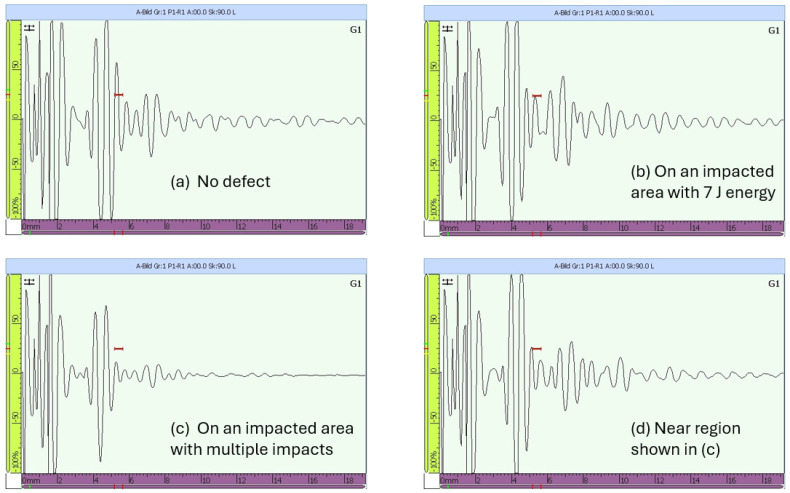
A-scans in Section 1.

**Figure 31:**
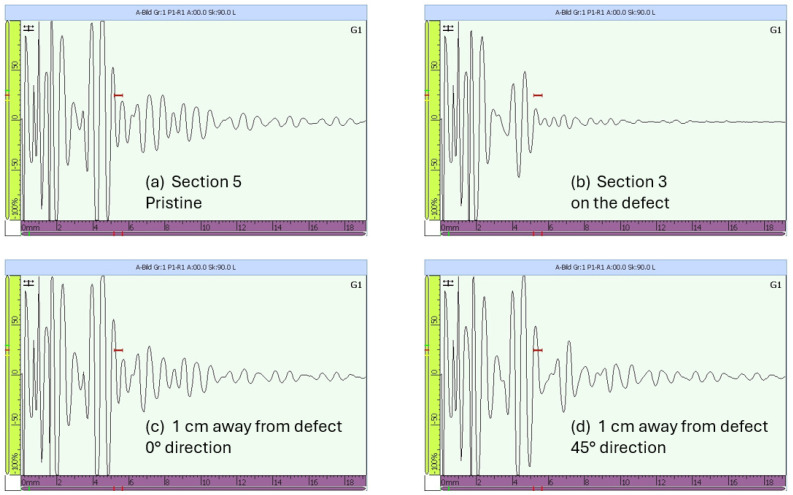
A-scans in Sections 3 and 5.

A visual inspection using the OmniScan MX2 was performed in Sections 1, 3, and 5 to evaluate and possibly confirm the presence of damage using a classical ultrasonic method. The scanning was performed manually and locally on small areas of the structure. It serves to confirm that operating such tools for hotspots on large airplanes is very time-consuming. To use it as a tool to confirm the presence of damage after having applied a global method would be ideal. The equipment was mounted with an ultrasonic phased array probe which introduced a pulse through the thickness to interrogate the structure. The same probe was used to record the wave traveling through the media in such a pulse-echo mode. The A-scan signals obtained in Section 1 and Sections 3 and 5 are presented in Figure 30 and Figure 31, respectively.

In pristine condition, two main waveforms are expected back to the probe: a first noisy signal along with a front wall echo from the contact surface (i.e., the echo is due to the wave excited by the piezoelectric crystal and scattering at the contact surface), and a second back-wall echo reflected from the opposite surface (i.e, the wave transmitted across the first surface travels through the composite media, is reflected from the opposite surface, and travels back to the probe). The back wall echo is mostly affected by the material properties, as the wave travels through the thickness twice before being recorded by the probe. When the material deteriorates, the wave is attenuated more, and the back wall echo either shows a lower amplitude or disappears. This is so in the case of delamination when some weak waveforms are reflected at the damage back to the probe, and the back wall echo is not visible anymore as the wave cannot travel across the delamination [45]. Instead, when a weak deterioration is present, the damage increases the attenuation, and the back-wall echo becomes weaker. This latter phenomenon is visible in Figure 30.

In the first case, when no defect is present, (a) the front- and back-wall echoes show similar amplitudes and are followed by several waveforms, which represent further scattering in between the plate thickness. The same applies after a very low impact is carried out (b), i.e., the energy is too low to enable any damage onset. Otherwise, after multiple impacts are applied to the same location (c), the amplitude of the back-wall echo decreases, and the following waveforms are strongly attenuated as well, i.e., the region is affected by an early emerging defect even though there is no visibility of the damage. The presence of minuscule damage is confirmed when moving the probe very close to the impact location (d) and repeating the ultrasonic inspection. In this case, the same outcome as in the case of no defect (a) is obtained.

Having a closer look at the A-scan signals obtained while inspecting Section 3 and Section 5, a very similar discussion arises (see Figure 31). Again, a very small defect is assessed (b) while no damage is visible either in pristine condition (a) or 1 cm away from the impact location along different directions (c, d). Indeed, a weak back echo is shown at the impacted spot in Section 3. In addition, the surface again does not show any modification to the naked eye.

**Figure 32:**
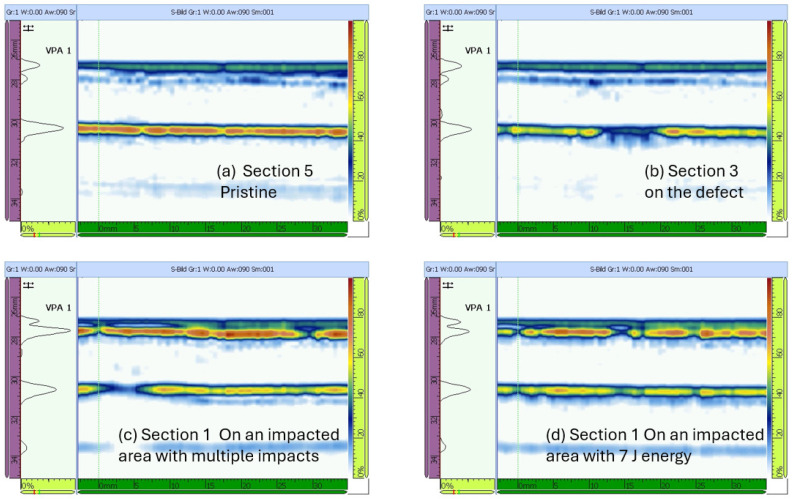
B-scans in Sections 1, 3, and 5.

In the B-scans, the entry signal is observed at 28 mm and the output signal at 30.5 mm (see Figure 32). The difference between these two values corresponds to approximately the structure thickness. The 34 mm scale indicates which area is being looked at (in depth). Therefore, the integrated wedge (the transparent plastic part under the test head) stops at approx. 27 mm, and the CFRP starts at around 28 mm.

The damage in Section 3, when the phased array was placed exactly on the spot where the multiple high-energy impacts were made, was able to be well detected. The back-wall echoes are much weaker, unlike in Section 5 (pristine state) or in Section 1 (on the spot with a low-velocity 7-joule impact), where the back-wall echoes were strong and uninterrupted. The back-wall echoes are still different between Section 5 (the pristine state) and Section 1 (a spot with a small impact but no damage).

On the left side of the scale, the envelope of the A-scan can be seen. If there were to be a significant delamination that causes a discontinuity in the lay-up, a peak in the middle would appear. The color scale shows a change in color from orange to blue when a small disturbance through the thickness is present.

The A- and B-scans represent a validation of the damage detection method based on guided wave measurements. The described damage did not cause a perforation in the structure at any point.

At this damage level, resulting from multiple impacts, with the highest energy being equal to 45 joules, a modification in the structural system occurred. However, these discontinuities are not responsible for a hazard that is endangering the structural integrity of the fuselage part. This stage of early damage proves the sensitivity of the Lamb-wave-based method.

## 7. Discussion

The experimental investigation showed that the wave velocity determined using analytical solutions is generally higher than what is determined using the recorded experimental data. This is mostly due to the transient phenomenon associated with wave propagation, which cannot be exactly met analytically. Hence, it is important to consider this when dealing with the design of the SHM system based on guided waves. In addition, it is worth noting how the presence of barely visible damage in the investigated structure did not always slow down the wave propagation. The wave propagation was assessed with wave path distances up to 840 mm and going along and through stiffeners. Excellent reproducibility of velocity calculations from the measured signals on two different skin sections of the panel was proven, as shown in Section 6.2. Indeed, adequate set-up parameters for data collection were defined to perform an extensive measurement campaign for the guided ultrasonic wave inspection, relying on a one-sided surface excitation adequate for aircraft NDT applications.

The damage caused by the impacts on the inner part of the panel was detected using both traditional methods and Lamb-wave-based measurements. For greater damage, according to the literature, delamination can lead to an increase in amplitude, whereas other damage types may lead to amplitude reduction [45]. In this investigation, a reduction in the wave amplitudes was not significant in the guided wave signals. In fact, the impacts performed at different energy levels, up to 45 joules, do not cause high-risk damage that endangers the structure’s integrity. This is demonstrated by classical ultrasonic testing, whose non-destructive evaluation showed very early damage present in the structure.

The tomographic reconstruction method, based on guided wave measurements and multi-parameter diagnosis while varying the distribution action over the structure, was applied to two different areas of the stiffened panel.

One area (Sections 1 and 2) was instrumented with 25 sensors (17 sensors on Section 1), and the highest impact energy was caused by 20-joule impacts. The inspection in Sections 1 to 2 of the structure allowed assessing the damage index most adequate to localize the impacted skin area precisely. No damage indicators based on amplitudes and time of flight were used due to the diversity of wave paths (i.e., the same input parameters were used for all paths, even though they had different sensor–actuator distances and these paths might have needed different actuation voltages). Indeed, the signals for some paths were too weak, and for other paths, high crosstalk was observed. Therefore, relying on wave parameters other than the amplitude is more effective. Considering the problematic type of early-stage BVID, a dense network with wave paths varying from 55 to 421 was used for this use case. The visualizations of the damage probability index algorithm showed that (1) the energy-based index and a linear-weight distribution function allow damage localization with 421 paths and with the sharpest DPI reconstruction when a 50% threshold is applied, (2) the RMSD-index allows precise localization and provides sharp imaging with both 421 paths and 55 paths, (3) cross-correlation diagnosis is possible but with a wide area of damage iso-probability, (4) the multi-parameter approach with a modified-linear weight function returns a sharp DPI reconstruction, and (5) when a reduced number of paths is used (i.e., 55 paths), only the RMSD-index localizes the impacted area correctly. Therefore, as explained in Section 6.8, this inspection assessed the effects of the number of wave paths on a damage index and the effects of the weighting function and threshold on achieving a sharp tomographic reconstruction.

The second area of the panel (Sections 3 to 5) was impacted by energy impacts of 30 and 40 joules. The final and highest impact of 50 joules was performed on the outer shell of the panel. The sensors were positioned on both sides of the stringers at strategic positions consistent with the lay-up directions. However, the Lamb wave method was insufficient to localize the damage, with only six or ten sensors placed apart from the stringers in this area. The intersections of wave paths with such minor damage might be limited to performing a reliable damage detection process. The increase in the number of wave paths reinforces the damage localization as well as the fusion of health indicators, as proven in Section 6.8. A second step was taken, where the number of sensors was increased from 10 to 25 to ensure that more wave paths were realized.

In Sections 3 to 5, the damage probability index algorithm showed the following: (1) Damage detection using ten sensors (Sensors 1 to 10 in Network 2, 53 paths) sparsed through three sections was not possible. (2) An unambiguous localization of the damage using the same damage indicators as for Section 1 was less evident, but the impacted area was correctly identified using the energy-based index, the RMSD-based index, and the multi-parameter approach. Indeed, the damage and the reflections from the stringers might have been, in this use-case, more complex than for Section 1. (3) All three indices detect the impacted area, but only the multi-parameter approach shows a high DPI around the hotspot. Still, detection is possible with no false alarms.

The energy-based damage index proved to be reliable for both investigated networks in locating the damage, provided a sufficient number of wave paths crossed the impacted area.

In addition, the new reference design model introduced in this paper, where Sections 3 and 5 of the panel are mirrored, indicates a phase shift between signals recorded in the pristine section and the mirror section. Although there are minor differences in the gluing process induced by human errors, the waveforms are similar and can still be used to extract information about the main physics of wave propagation.

It was not proven that a sensor’s size, equal to half the wavelength of the ultrasonic wave traveling in the structure, has a direct influence on the detection and localization of barely visible damage. This study contributes to providing an experimental proof of concept to qualify the guided waves method as an SHM approach for the primary structure. By examining the guided wave data and establishing data-analysis protocols for a particular use case, we ensure that robust measurements can be taken and that promising results for validation work can be obtained.

## 8. Conclusions

In this paper, we carried out major experimental work and comprehensive evaluations to physically test an SHM aerospace system using a fuselage segment.

Investigations related to wave propagation and damage detection were performed, and a number of major conclusions and assessments can be drawn. In particular, to ensure safe flight systems and improve performance, it is vital to achieve high endurance during the structural design phase as well as integrate SHM technologies in the aircraft design process (and during scheduled maintenance). This experimental study on a stiffened composite structure presented real data obtained from networks of soft piezoelectric sensors. The first part involved understanding the wave propagation in a structure with partially unknown properties, the types of modes that can be detected, and the sensor type and configuration to consistently record wave signals. Finally, several impact energy levels were performed, and a damage detection process was developed and optimized with a varying number of wave paths.

Experimental estimation of the group velocities of the fastest mode was realized using two different networks of piezoelectric sensors, and a dense network of 25 sensors sparsely arranged facilitated the detection of barely visible damage using 55, 292, and 421 paths.

Experimentally, via piezoelectric transducers, it was shown that the damage detection process is directly related to the coverage density of the structure and number of sensor–actuator optimal paths crossing the damaged spot, regardless of sensor positioning in the strategic lay-up directions. In this way, we assessed the degree to which the mitigation of sensor distribution can detect and localize barely visible impact damage. The best DPI image reconstruction was achieved in Section 1 with the following damage indices and weighting distribution function: energy-based index with 421 paths and 50% threshold, RMSD index with 55 and 421 paths, multi-parameter approach with a modified linear distribution function and 421 paths. In Section 3, a fusion of the health indicators reinforced the damage detection process algorithm. Indeed, the localization of the impacted area was possible with the energy-based and RMSD indices. Still, a multi-parameter approach with up to 292 paths provided better hotspot localization.

Overall, this study is an experimental demonstration of the feasibility of using Lamb waves as a global diagnostics tool for barely visible damage detection in a large-scale anisotropic fuselage structure. It provided experimental data from real aircraft-grade material and investigated the type of sensor configurations possible for tuned Lamb-wave excitation and detection for both wave-propagation studies and structural health monitoring.

## Data Availability

Data are contained within the article.

## Abbreviations

The following abbreviations are used in this manuscript:

AE	Acetic acid Ethyl Ester
ADC	Analog to Digital Converter
BNC	Bayonet Neill–Concelman
BVID	Barely Visible Impact Damage
CAI	Compression After Impact
CC	Correlation Coefficient
CFRP	Carbon Fiber-Reinforced Polymers
CG	Center of Gravity
CS	Certification Specification
CT	Computed Tomography
CV	Coefficient of Variation
DAC	Digital to Analog Converter
DAS	Delay-and-Sum
DI	Damage Index
dI	Distance Index
DLR	German Aerospace Center
DPI	Damage Probability Index
EASA	European Union Aviation Safety Agency
FAA	Federal Aviation Administration
FBG	Fiber Bragg Grating
FFT	Fast Fourier Transform
FIR	Finite Impulse Response
GLARE	GLAss REinforced aluminum laminates
GW	Guided Wave
IDT	Interdigital Transducer
MRD	Minimum Resolvable Distance
MS	Mega Samples
MVDR	Minimum Variance Distortionless Response
NASA	National Aeronautics and Space Administration
NDE	Nondestructive Evaluation
NDI	Non-Destructive Inspection
NLR	Netherlands Aerospace Centre
OFDR	Optical Frequency Domain Reflectometry
ONERA	The French Aerospace Lab
PEEK	Polyetheretherketone
PDI	Damage Probability Index
PWAS	Piezoelectric Wafer Active Sensors
PZT	(Pb—lead, Zr—zirconium, Ti—titanium)
RAPID	Reconstruction Algorithm for Probabilistic Inspection of Damage
RMSD	Root Mean Square Deviation
RMSE	Root Mean Square Error
RTM	Resin Transfer Moulding
SAW	Surface Acoustic Wave
SD	Standard Deviation
SHM	Structural Health Monitoring
SMART	Stanford Multi-Actuator–Receiver Transduction
TFM	Total Focusing Method
TUM	Technical University of Munich
USB	Universal Serial Bus
UT	Ultrasonic Testing
XWB	Xtra Wide-Body

## Appendix A

The theoretical and experimental drop velocities, the impactor weight used in the impact set-up, and the measured kinetic energy are summarized in Table A1.

**Table A1:** Impact energy calculations and measurements.

Impact Energy [J]	Mass [Kg]	Height [mm]	Theoretical Drop Velocity [mm/s]	Experimental Drop Velocity [mm/s]	Experimental Kinetic Energy [m/s^2^]
1	2.49	41	897	725	0.65
2	3.8	53	1026	–	–
2	2.49	82	1291	1090	1.48
5	2.49	204	2001	2023	4.98
5	3.8	134	1624	–	–
7	2.49	287	2373	2260	6.36
10	2.49	409	2833	2685	8.98
20	2.49	819	4008	3889	18.83
25	2.49	1024	4481	4270	22.70
30	2.49	1228	4909	4242	22.41
30	4.3	710	3763	3725	29.50
40	4.3	950	4317	4270	39.20
45	4.3	1067	4575	4329	40.30
50	4.3	1190	4832	–	–

## Appendix B

The sensor properties and the network compositions are summarized in Table A2, Table A3 andTable A4.

**Table A2:** Physical and piezoelectric properties of the sensors.

Properties	Units	PIC151	PIC255	PZT-5B
Diameter	mm	10	10, 6.5, 5	15
Dimension	mm	−−	10 × 10	−−
Thickness (disc)	mm	0.5	0.2, 0.3, 0.35, 1	0.22
Thickness (shear)	mm	−−	3, 0.5	−−
Density	g/cm^3^	7.8	7.8	7.5
$d_{31}$	$10^{-12}$ C/N	−210	−180	−210
$d_{33}$	$10^{-12}$ C/N	500	400	480
$g_{31}$	$10^{-3}$ m^2^/C	−11.5	−11.3	−10
$g_{33}$	$10^{-3}$ m^2^/C	22	25	24.5
$N_{p}\;\left(planar\right)$	Hz·m	1950	2000	1990
$N_{t}\;\left(thickness\right)$	Hz·m	1950	2000	2100
$Y_{11}^{E}$	$10^{10}$ N/m^2^	10	15.6	7.4

**Table A3:** Sensor network number 1.

Sensor Number	Diameter [mm]	Thickness [mm]	Material
1 to 7	10	0.5	PIC151
8 to 10	10	1	PIC255
11	10	0.35	PIC255
12	10 × 10	3	PIC255
13 to 16	10	0.35	PIC255
17	6.5	0.3	PIC255
18	10	0.5	PIC151
19	10	0.5	PIC151
20	10	0.35	PIC255
21	10	0.35	PIC255
22	10	0.2	PIC255
23	10	0.2	PIC255
24	10	1	PIC255
25	10	0.35	PIC255

**Table A4:** Sensor networks number 2 and 3.

Sensor Number	Diameter [mm]	Thickness [mm]	Material
1 to 6	10	0.5	PIC151
7	10 × 10	0.2	PIC255
8	5	0.2	PIC255
9	5	0.2	PIC255
10	15	0.22	PZT-5B
11 to 16	10	0.5	PIC151
17	10	1	PIC255
18	10	0.5	PIC151
19	10	0.35	PIC255
20 to 24	10	1	PIC255
25	15	0.22	PZT-5B
